# Economic evaluations of screening strategies for the early detection of colorectal cancer in the average-risk population: A systematic literature review

**DOI:** 10.1371/journal.pone.0227251

**Published:** 2019-12-31

**Authors:** Joan Mendivil, Marilena Appierto, Susana Aceituno, Mercè Comas, Montserrat Rué

**Affiliations:** 1 Outcomes Research and Epidemiology, Shire International GmbH, a Takeda Company, Zug, Switzerland; 2 Health Economics department, Outcomes’ 10 SLU, Castellon, CS, Spain; 3 Epidemiology and Evaluation Department, IMIM (Hospital del Mar Medical Research Institute); Red de Investigación en Servicios de Salud en Enfermedades Crónicas (REDISSEC), Barcelona, Spain; 4 Departament of Basic Medical Sciences, Universitat de Lleida, Lleida, Spain; University Magna Graecia of Catanzaro, ITALY

## Abstract

**Background:**

Colorectal cancer (CRC) screening has proven effective in reducing CRC mortality. This study aimed to systematically review, and evaluate the reporting quality, of the economic evidence regarding CRC screening in average-risk individuals.

**Methods:**

Databases searched included Medline, EMBASE, National Health Service Economic Evaluation, Database of Abstracts of Reviews of Effects, Cost-Effectiveness Analysis registry, EconLit, and Health Technology Assessment database. Eligible studies were cost-effectiveness and cost-utility analyses comparing CRC screening strategies in average-risk individuals, published in English or Spanish, between January 2012 and November 2018. Reporting quality was assessed using the Consolidated Health Economic Evaluation Reporting Standards (CHEERS) checklist.

**Results:**

Of 1,993 publications initially retrieved, 477 were excluded by duplicate review, 1,449 by title/abstract review, and 34 by full-text review. Finally, 33 publications were included in the qualitative synthesis. Most studies were conducted in Europe (36,4%), followed by United States (24,2%) and Asia (24,2%). The main screening modalities considered were fecal immunochemical tests (70%), colonoscopy (67%), guaiac fecal occult blood test (42%) and flexible sigmoidoscopy (30%). In most studies, CRC screening was deemed cost-effective compared to no screening. Sensitivity analyses indicated that cost of CRC screening tests, adherence to screening, screening test sensitivity, and cost of CRC treatment had the greatest impact on cost-effectiveness results across studies. The majority of studies (73%) adequately reported at least 50% of the items included in the CHEERS checklist. Least well reported items included setting, study perspective, discount rate, model choice, and methods to identify effectiveness data or to estimate resource use and costs.

**Conclusions:**

CRC screening is an efficient alternative to no screening. Nevertheless, it is not possible to conclude which strategy should be preferred for population-based screening programs. Although we observed an overall good adherence to CHEERS recommendations, there is still room for improvement in economic evaluations reporting in this field.

## Introduction

Colorectal cancer (CRC) is the third most common cancer worldwide and it is associated with high morbidity and mortality [1]. According to the World Health Organization, in 2018, CRC accounted for approximately 1.8 million new cases and 880 792 deaths globally, being the second cause of cancer-related death [1].

In early stages, CRC is mainly asymptomatic, and most CRCs are diagnosed after symptoms onset, when the disease is already at an advanced stage and has a poor prognosis [2,3].

CRC screening in asymptomatic individuals has been shown to reduce the incidence and mortality of CRC, allowing the identification and removal of preneoplastic lesions, as well as, increasing the rate of diagnosis at an early stage [4,5]. Based on this, several countries worldwide have implemented screening programs to promote early detection of CRC in the average-risk population (asymptomatic individuals, with no personal nor family history of CRC) [6].

Indeed, most guidelines recommend that individuals at average risk of CRC should submit to CRC screening [7]. Nevertheless, it is possible to observe a certain disparity across CRC screening recommendations worldwide [7]. In the United States, for instance, the most recent recommendations from the U.S. Multi-Society Task Force on Colorectal Cancer identify colonoscopy every 10 years or annual fecal immunochemical test (FIT) as the preferred tests for average risk individuals screening between 50 and 75 years [8]. On the contrary, in Canada, screening is recommended in adults aged 60 to 74 years and screening modalities suggested are fecal occult blood test, with either guaiac fecal occult blood test (gFOBT) or FIT, every two years, or flexible sigmoidoscopy (FS) every 10 years, while colonoscopy is not recommended as a screening test [9]. In Europe, The European Colorectal Cancer Screening Guidelines Working Group recommends screening individuals between ages 50 and 74, identifying the gFOBT/FIT (1–2 years) as the preferred approach and highlighting that, as of 2013, FOBT was the only screening method approved throughout the European Union [7]. It must be noted that due to differences in the availability of the various CRC screening test, recommendations for screening also vary between countries (e.g. German guidelines recommend using colonoscopy every 10 years in adults ≥ 50 years, while Spanish guidelines suggest using FIT every 2 years as preferential approach) [7]. Likewise In Asia, main recommendations differ across countries [7]. For example, the Chinese Society of Gastroenterology recommends individuals between ages 50 and 74 to undergo FOBT, followed by a questionnaire (every 3 years) to identify high-risk factor, while in Japan the approach to CRC screening is based on the use of FIT, among individuals aged 40–69 years [6,7].

Thus, although FOBT, FS or colonoscopy are the most frequently recommended screening modalities across guidelines, there appears to be no single preferred screening test, and the choice should be based, among other factors, on local resource availability, expected adherence to screening and surveillance requirements [7].

Of note, despite the more limited evidence supporting their use, the availability of less-invasive screening modalities such as CT colonography, video capsule colonoscopy, stool DNA (sDNA) and blood DNA testing, together with the absence of specific recommendations regarding their use in most clinical guidelines, further complicates screening modality selection for policymakers [10]. Besides the necessity of high-quality clinical evidence given the cost of population-based screening programs, the availability of high quality—and transparent reporting of- economic evidence is also essential to inform decision-making on the selection of a specific screening strategy and ensure efficient resources allocation. In particular, cost-utility and cost-effectiveness analyses provide valuable information regarding which, among two or more alternatives may provide the best value for money, and thus, are helpful tools to prioritize among different health care interventions [11].

Despite this, recent research has shown that a high proportion of economic studies in several fields, lack transparency and do not have an optimal reporting quality [12–15]. In a previous work, Jeong et al. systematically reviewed and provided a critical appraisal of the methodological approaches used in cost-utility and cost-effectiveness analyses of CRC screening published in English up to 2012 [16]. Nevertheless, following the approval in the latest years of new CRC screening tests, such as multi-target stool DNA test, methylated septin 9 assay etc., it seems necessary to provide an update of the economic evidence available in the field [10]. Thus, the aim of the present work was to review the economic evaluations of CRC screening test, to verify the degree and reporting quality of evidence generated for currently available CRC screening test/approaches. To do so, we defined four specific objectives: 1) identify studies examining the efficiency of CRC screening tests, in view of their potential use for average risk individuals screening; 2) evaluate their reporting quality; 3) determine CRC screening tests for which there is still limited evidence regarding their efficiency; 4) identify main parameters/inputs influencing economic evaluations results.

## Methods

In order to fulfill the aforementioned objectives, we formulated the following research questions:

1. ¿Is there new economic evidence examining the efficiency of currently available CRC screening tests, in view of their potential use for average risk individuals screening?
2. ¿Is the reporting-quality of the economic evaluations available optimal?
3. ¿What are the CRC screening techniques that have been less frequently evaluated in economic studies?
4. ¿What are the main parameters influencing economic evaluations results?

A review protocol was developed (S1 Text) and a systematic literature review was conducted in accordance with the Preferred Reporting Items for Systematic Reviews and Meta-Analyses (PRISMA) guidelines [17] (S1 File).

### Literature search

A systematic search was conducted on November 22, 2018 in the following databases: MEDLINE (via Ovid: 2012–22.11.2018), EMBASE (via Ovid: 2012–22.11.2018), Cost-Effectiveness Analysis (CEA) registry (2012–22.11.2018), National Health Service Economic Evaluation Database (NHS EED) (via CRD: 1.01.2012–22.11.2018), Database of Abstracts of Reviews of Effects (DARE) (via CRD: 1.01.2012–22.11.2018), EconLit (1.01.2012–22.11.2018) and HTA (via CRD: 1.01.2012–22.11.2018). Depending on the characteristics of the database, different search strategies were used (S2 Text. Search strategies). A hand-search of reference list of relevant articles was also conducted.

### Eligibility criteria

Full economic evaluations (cost-effectiveness and cost-utility analysis) comparing costs and clinical benefits of CRC strategies, for the early detection of CRC, were selected. Studies had to fulfill the following criteria:

- Population: since main guidelines recommend a population-based approach, among average-risk individuals, for CRC screening [7], we limited our search to full economic evaluations conducted in the general, average-risk population (studies including either screening naïve members of the general population, general average-risk population or asymptomatic members of the general population were selected).
- Intervention: CRC screening
- Comparator: alternative CRC screening strategy or no screening
- Outcome: cost per quality-adjusted life-year (QALY); or cost per life-year gained/saved (LYG/LYS)
- Language: English or Spanish.
- Publication date: between January 1, 2012 and November 22, 2018.

Since the objective of the study was to identify studies examining the efficiency of CRC screening tests, we excluded cost studies, cost of illness, disease burden studies, and clinical studies, as they do not provide information regarding the relationship between cost and outcomes of the CRC strategies evaluated. Letters to the editor, editorials, abstracts and conference proceedings as well as narrative and systematic literature reviews were also excluded as they may not provide sufficient details to allow for individual studies evaluation.

Since the objective of the study was not to develop specific recommendations regarding CRC strategies to be implemented, but to verify the degree and reporting quality of evidence generated for currently available CRC screening test/approaches, no limitation was applied to either the CRC screening tests or study settings (i.e. a specific country or continent) to be included.

### Selection of studies and data extraction

Following duplicates removal, titles and abstracts were screened to identify relevant publications. Eligibility was then assessed by full text review. Two independent researchers (JM, MA) screened the studies based on preset inclusion and exclusion criteria. Discrepancies were resolved by consensus. Relevant data from each selected publication were extracted into an extraction form including year and country of the study, study perspective, time horizon and cycles, population, interventions and comparators, modelling approach, effectiveness data sources, outcome measures, costs, cost data sources, year of costing, inflation adjustment, discount rate, reported results, sensitivity analysis, key variables influencing results, reported limitations, model validation and conclusions.

### Assessment of quality of reporting

Reporting bias and overall reporting quality was assessed using the Consolidated Health Economic Evaluation Reporting Standards (CHEERS). The CHEERS statement is aimed to optimize reporting quality of health economics evaluations; it consists of a 24-items checklist, covering six main categories: title and abstract, introduction, methods, results, discussion, source of funding, and conflicts of interest [18]. Reporting of each item was assessed indicating “adequately reported” when recommendations were fully met, “inadequate reporting” when they were not completely fulfilled, “not reported” when they were not fulfilled and “not applicable” when reporting of the item was not required in the analyzed study. Overall adherence to CHEERS statement was also assessed for each selected study as the percentage of adequately reported items. To avoid possible between-assessor variability in the quality assessment of included studies, the reporting quality of all studies was evaluated by the same researcher (MA).

### Bibliometric analysis

In order to determine CRC screening tests for which there is still limited evidence regarding their efficiency, as well as, parameters most frequently reported to influence study results, we conducted a bibliometric analysis of included studies. CRC screening techniques evaluated, and influential parameters were manually extracted from included studies. Data extracted were analyzed using VOSviewer (v. 1.6.5), a software tool for creating maps based on network data and for visualizing and exploring these maps [19]. A network visualization map (items are represented by nodes) was generated to visualize occurrence and co-occurrence of CRC screening strategies evaluated across studies [19]. In the network visualization map, CRC screening techniques are represented by a label and a node (circle) [19]. The size of the node for each CRC strategy in the map represents its frequency of occurrence across retrieved studies [19]. Larger node size indicates a high frequency of occurrence [19]. Lines between nodes indicate co-occurrence, indicating that CRC screening strategies represented were evaluated in the same study [19]. Position of the nodes in the map indicate their relatedness: the smaller the distance between two nodes, the higher their relatedness [19].

Occurrence and co-occurrence of parameters influencing the results were analyzed by generating a density visualization map (items are represented by a label and group by colors) [19]. In the map, influential parameters identified across studies are represented by a label and grouped by color [19]. Parameters with higher frequency of occurrence are represented in red and a bigger font size is employed for the label. Conversely, parameters with less frequency of occurrence are depicted in blue and with a smaller font size [19]. Similarly to the network visualization map, the distance between labels indicate the relatedness of the parameters [19]. The smaller the distance, the higher the relatedness, indicating that parameters that are located closer in the map, frequently co-occur in the same study [19].

In order to facilitate the interpretation of the bibliometric analyses, variations in screening starting age and screening intervals of the same CRC screening technique were grouped under the same label (e.g. colonoscopy every 10 years and colonoscopy once only at 65 years were grouped under the label colonoscopy [COL])(S3 Text). A full description of CRC strategies evaluated across studies is presented in supplementary material (S1 Table). With the same purpose, words referring to the same influential parameters were grouped under the same label (S3 Text).

## Results

### Study selection

**Fig 1:**
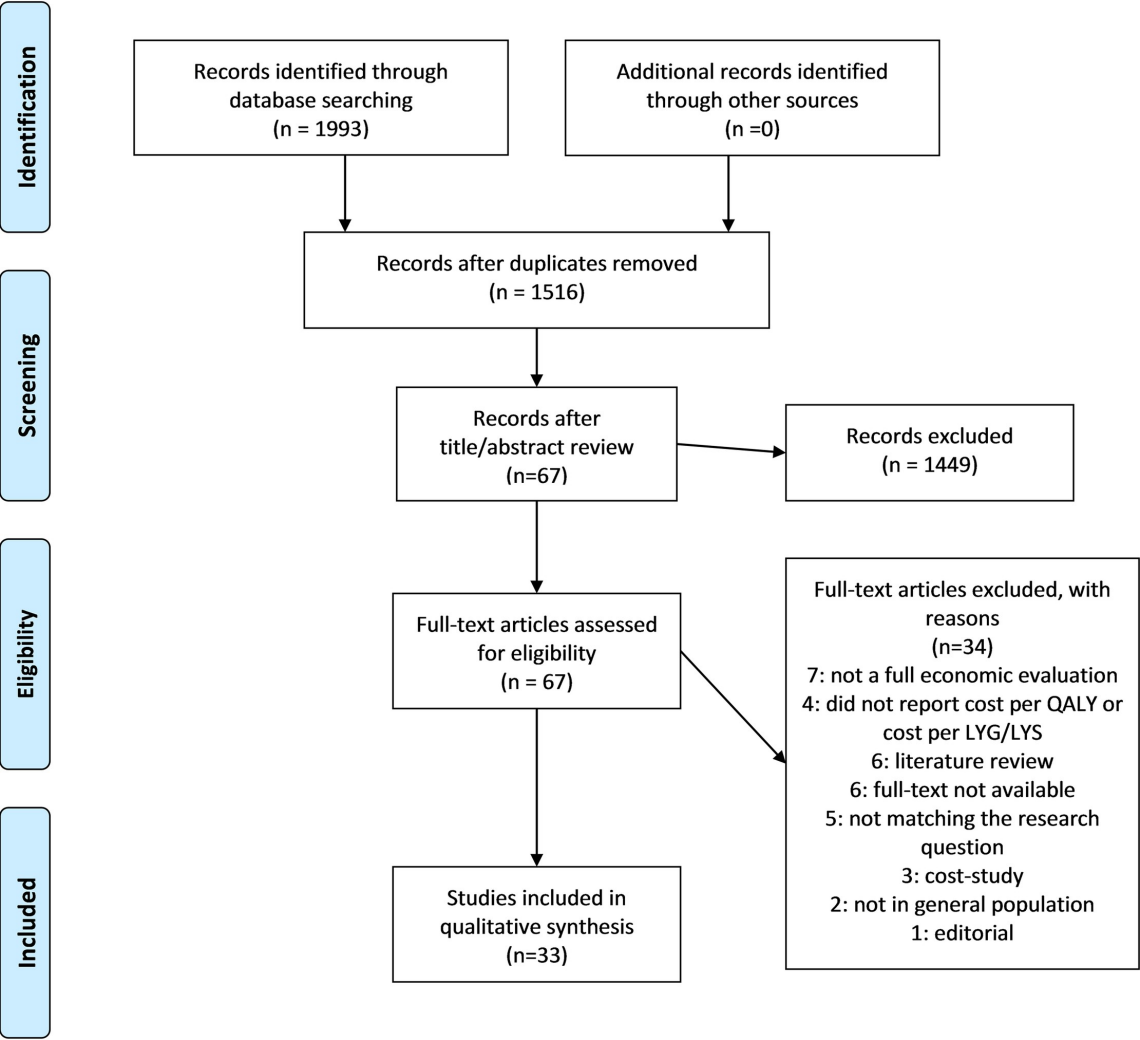
Flow diagram of publication selection according to PRISMA guidelines.

The search yielded 1993 references. Following duplicates removal (n = 477), publications were screened by title and abstract. A total of 1449 publications were excluded as they did not meet inclusion criteria. The full texts of the remaining 67 articles were assessed for eligibility. Of them, 33 were included in the qualitative synthesis. A flow diagram of the selection process, according to the PRISMA Guidelines is depicted in Fig 1.

### Study characteristics

Main characteristics of included studies are presented in supporting material (S1 Table).

#### Setting and population

Most of the studies were conducted in Europe (n = 12; 36,4%) [20–31], followed by United States (n = 8; 24,2%) [32–39] and Asia (n = 8; 24,2%) [40–47]. Of the remaining studies, two (6,1%) were conducted in South America [48,49] two in Canada [50,51] and one in Australia (3%) [52]. In line with the scope of the review, the population included in the studies consisted of individuals at average risk of CRC, from the general population (naïve members of the general population, general average-risk population or asymptomatic members of the general population). Further details regarding the population included in the selected studies are presented in supporting material (S4 Text).

As for the characteristics of the population, most of the studies (n = 28; 85%) included subjects 40 years or older, while the remaining five studies included subjects 20 (n = 3; 9,1%) [27,48,52] or 30 years old (n = 2; 6,1%) [23,31]. In most of the studies, subjects were eligible for screening between 45 and 75 years. Nevertheless, several studies aimed at identifying the optimal age for CRC screening, thus different starting and stop age, as well as screening intervals, were simulated for each of the CRC screening strategy evaluated [20,23,27,28,30,31,34,36,37,40,47,48,50,52].

#### Time horizon

Over half of the included studies (n = 22; 67%) used a lifetime time horizon (simulation was stopped at maximum life expectancy or death, whichever occurred first) [20,21,23–31,38,39,41,42,45,46,48,50–52]. In eight (21%) of the remaining studies, time horizon varied between 20 and 50 years [22,34–36,40,43,44,49]. Four studies (12%) did not clearly state it [32,33,37,47].

#### Study perspective

Most of the studies used a health care payer (n = 14; 42,4%) [21,23–31,41,42,44,52] or a third-party payer perspective (n = 14; 42,4%) [20,22,34,36–39,43,45,46,48–51]; perspective was not reported in four studies (12,1%)[33,35,40,47]; while only one study used a societal perspective (3%)[33].

#### Interventions and comparators

**Fig 2:**
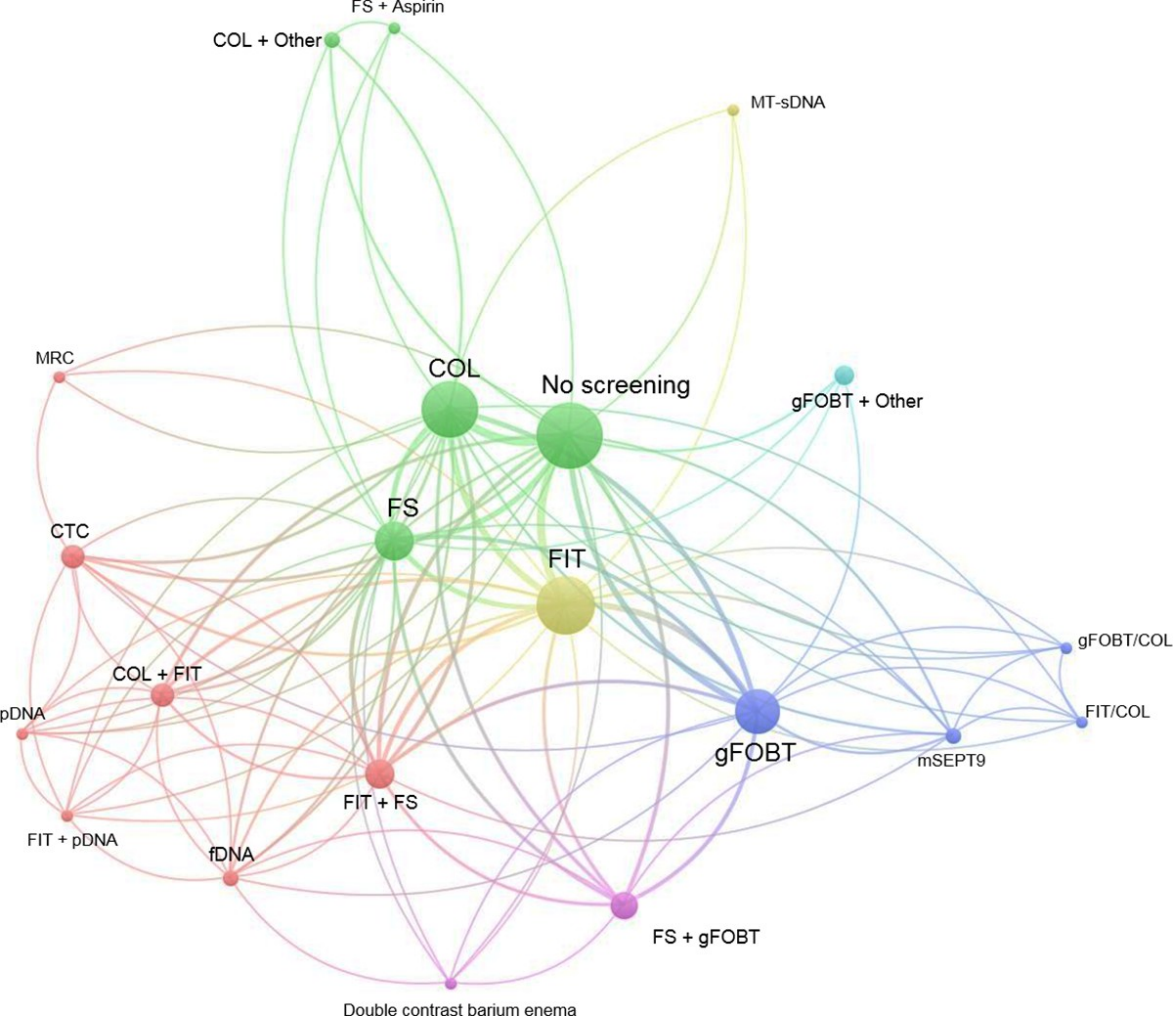
Bibliometric analysis of CRC screening strategies evaluated across studies. COL: colonoscopy; CTC: computed tomography colonography; fDNA: fecal DNA; FIT: fecal immunochemical test; FIT/COL: hybrid strategy with biennial FIT or COL twice; FS: flexible sigmoidoscopy; gFOBT: guaiac fecal occult blood test; gFOBT/COL: hybrid strategy with biennial gFOBT or COL twice; MRC: magnetic resonance colonography; mSEPT9: methylated septin 9 gene; MT-sDNA: multi target-stool DNA; pDNA: plasma DNA.

A wide range of CRC screening modalities were identified. Fig 2 shows the results of the bibliometric analysis of CRC screening strategies evaluated across studies.

Results are presented by means of a network visualization map. Based on the size of the nodes, we observed that the CRC screening tests most frequently evaluated across studies were FIT (n = 23; 70%); colonoscopy (COL; n = 22; 67%); gFOBT (n = 14;%; 42%) and FS (n = 10; 30%). Each of these four techniques was compared with no screening, which was the most frequently used comparator (n = 28; 85%). In addition, the distance between their nodes indicates that FIT, COL, gFOBT and FS where frequently compared with each other. The relatively smaller size of the corresponding nodes indicates that combinations of CRC screening techniques (e.g. COL+FIT; FIT+FS etc.), as well as more recently developed screening tests (i.e. Computed tomography colonography [CTC], fecal DNA [fDNA], multi target-stool DNA test [MT-sDNA], plasma DNA [pDNA] and methylated septin 9 gene [mSEPT9] testing were less frequently evaluated. Thus, there is still limited economic evidence regarding the use of these techniques for average-risk individuals screening.

#### Study designs and modelling approach

Most studies (n = 21; 64%) were cost-utility analyses, in which the cost-effectiveness ratio was calculated as incremental cost divided by incremental quality adjusted life years gained (QALYs). All the remaining studies (n = 12; 36%) consisted of cost-effectiveness analysis. Of them, seven reported incremental cost per life years gained [22,24,27,30,38,40,48], while five assessed incremental cost per life years saved [32,33,43,47,52].

The studies used different modelling techniques to evaluate the efficiency of the CRC screening modalities examined. State-transition modelling (i.e. Markov model) was the most common approach employed (n = 24; 73%). The second most frequent approach was microsimulation modelling, which was used in seven studies (21%) [20,24,27,30,50–52]. One of the studies used a decision analytic model, while the remaining one used the Archimedes model [30]. Briefly, the Archimedes model is a large-scale simulation model of human physiology, diseases, and health care system [53]. The model is based on a set of equations that represent physiological pathways of several conditions including CRC [53].

#### Characterizing uncertainty

All studies performed sensitivity analyses to account for data uncertainty. Most of the studies (n = 19; 58%) only performed deterministic sensitivity analyses (i.e. one-way, two-way, multiway and scenario sensitivity analyses). The remaining (n = 14; 42%) studies assessed uncertainty through both probabilistic and deterministic sensitivity analyses [22,23,26,28,36,38,40–42,44–46,48].

#### Cost-effectiveness results

Although a quantitative comparison between study results was not possible due to the heterogeneity in studies’ characteristics (i.e. settings, effectiveness outcomes chosen, modelling methods used, characteristics of the CRC screening strategies employed etc.), most studies indicated that CRC screening in average risk population might provide good value for money compared to no screening, independently of the screening modality chosen [20,21,23–25,27,28,30–43,45–52]. Due to the mentioned differences between studies, it was not possible to establish which CRC screening strategy would be the most efficient when implementing a population-based screening program.

#### Parameters influencing studies’ results

**Fig 3:**
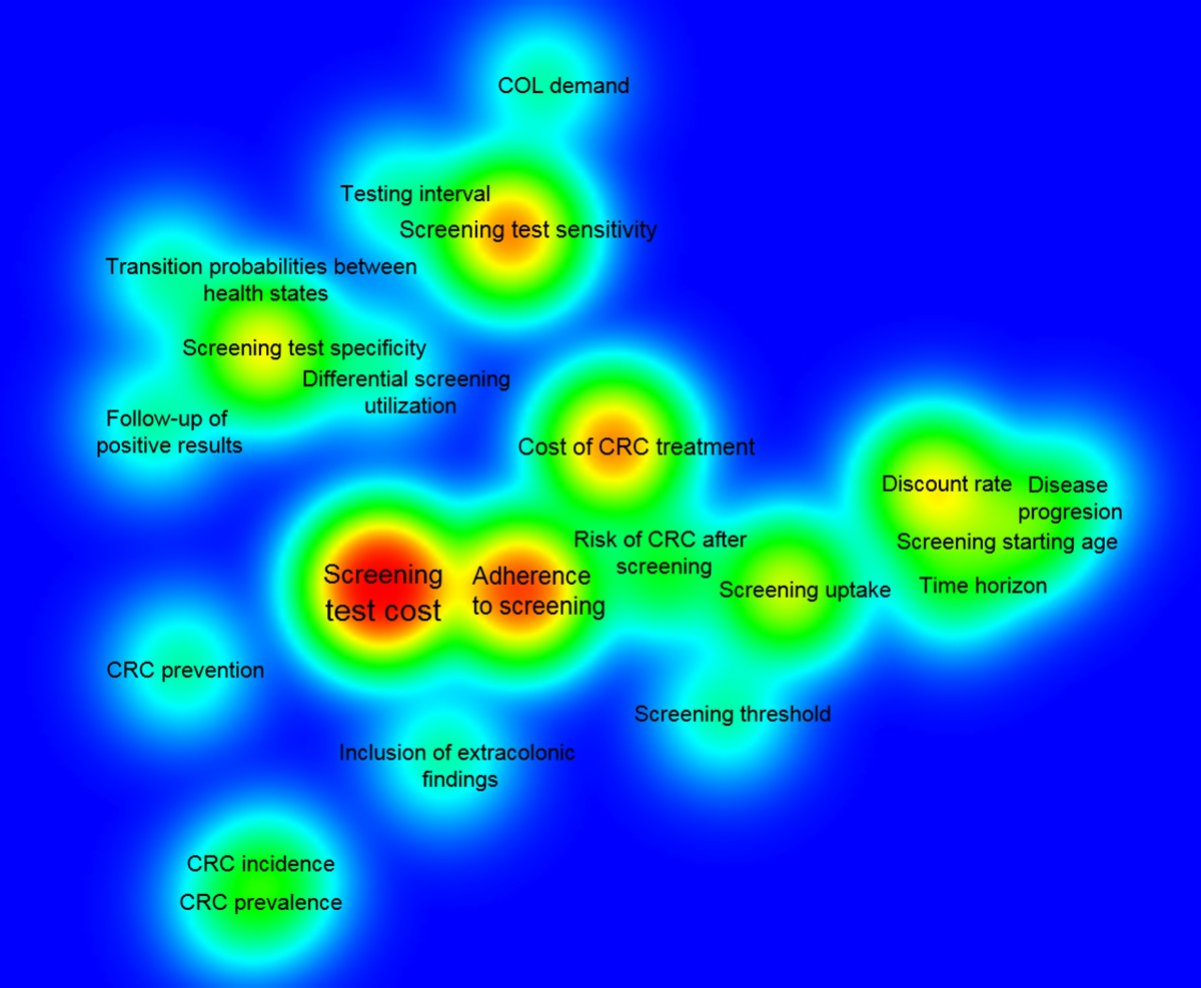
Bibliometric analysis of parameters influencing cost-effectiveness results across studies (density visualization map). COL: colonoscopy; CRC: colorectal cancer.

Fig 3 shows the results of the bibliometric analysis of parameters influencing cost-effectiveness results across studies. Results are presented by means of a density visualization map.

Based on the intensity of the color and the font size of the labels, we observed that, the parameters whose variation was more frequently reported to influence results across studies are screening cost (n = 19, 58%), adherence to screening (n = 12, 36%), screening test sensitivity (n = 9, 27%) and the cost of CRC treatment (n = 9, 27%). Interestingly, the relative position of the parameters in the map indicates that besides being the most frequently reported influential parameters, "screening test cost" and "adherence to screening" are also frequently co-occurring in the studies. This indicates that in most of the studies, variation in both parameters contributed to the uncertainty of the results.

### Assessment of reporting quality

**Fig 4:**
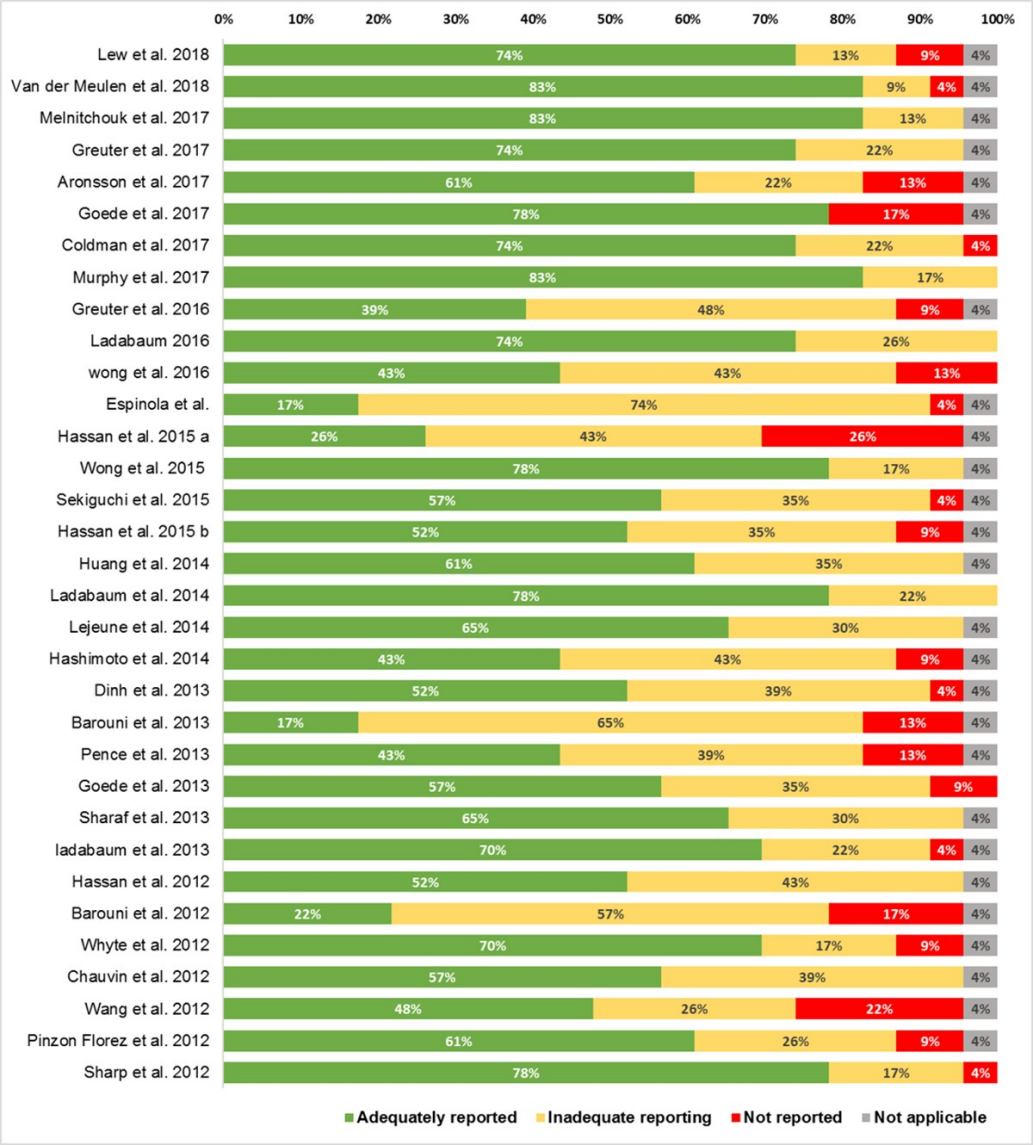
Adherence to CHEERS guidelines of selected articles.

Overview of adherence of selected articles to each of the CHEERS items is presented in supporting material (S1 Fig). Adherence to CHEERS guidelines of selected articles is depicted in Fig 4.

The majority of studies (n = 24; 73%) adequately reported at least 50% of the items included in the CHEERS checklist [20–23,25,26,28–32,34,36–39,41–43,48,50–52]. Despite not achieving at least 50% adherence to CHEERS recommendations, all the remaining studies (n = 9; 27%), reported at least 70% of the items included in the CHEERS, albeit incompletely [27,33,35,40,44–47,49].These data indicate that most of the aspects considered relevant according to the CHEERS recommendations are at least partly addressed in the studies.

As previously described, some of the studies assessed cost-effectiveness of CRC screening based on the evaluation of cost/LYS or LYG [22,24,27,30,32,33,38,40,43,47,48,52]. In addition, in all studies using preference-based outcomes (i.e. QALYs) as a measure of effectiveness, utility data were extracted from the literature. Thus, reporting of the item “measurement and valuation of preference-based outcomes”, was considered as not applicable and was not considered for the assessment of overall adherence to CHEERS recommendations.

**Fig 5:**
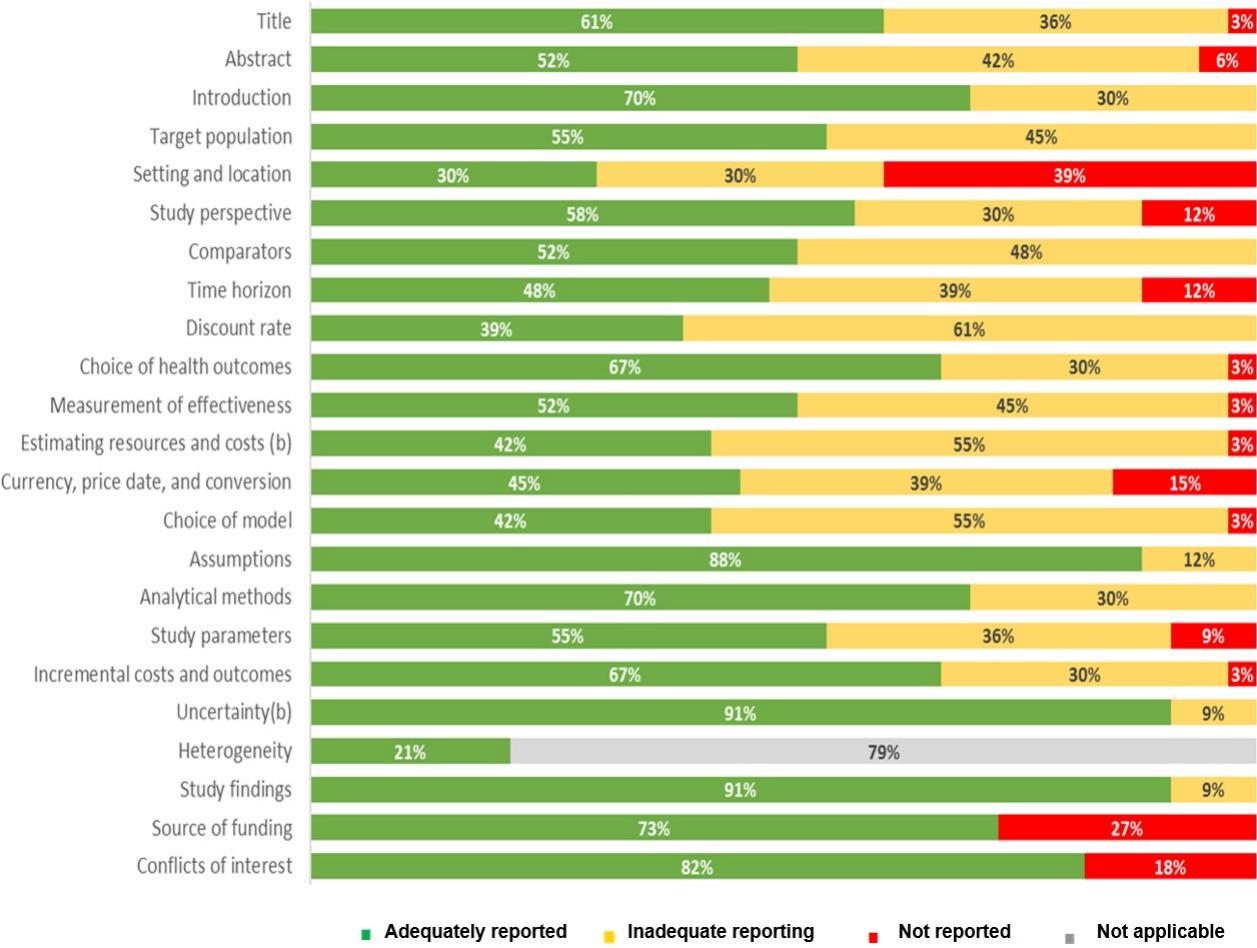
Reporting of CHEERS items across studies.

Reporting of single items across selected studies is depicted in Fig 5.

Most of the items (n = 16; 67%) were adequately reported in at least 50% of selected studies. Although most of the studies clearly complied with CHEERS recommendations regarding title reporting, approximately 36% of them (n = 12) [22,27,31,34,36,39,40,43–46,49] did not report the interventions being evaluated in the title, while one of them did not identify the study as an economic evaluation [50].

Among other factors, recent studies have suggested that efficiency of CRC screening may be influenced by the defined age range in which an individual is considered eligible for screening [54]. Thus, adequate reporting of target population characteristics is necessary to contextualize study findings and, when needed, to identify the optimal candidates for screening. All the studies, except one [49], reported baseline population characteristics (i.e. age, CRC risk etc.), nevertheless, only 55% of the studies (n = 18) justified their choice [20–22,24,26,28,29,31,32,36,38,39,42,43,45,50–52]. Regarding the reporting of setting and location, 39% (n = 13) of the studies did not state relevant aspects of the system in which the decision regarding the adoption of the CRC screening strategy had to be made. Only 30% (n = 10) of the studies adequately reported this item, complicating the interpretation of the study findings [21–24,28,31,37,39,43,51].

In economic evaluations, the choice of the study perspective strongly influences the study design, as it determines which cost components need to be accounted for in the analysis [55]. In the present review we observed that although most articles (n = 19; 58%) adequately reported study perspective, approximately 30% (n = 10) of them failed to relate it to the costs being evaluated [25,27,28,30,31,37,39,42,45,46]. Conversely, 12% (n = 4) of the studies did not report it [33,35,40,47].

Only 52% (n = 17) of the studies adequately reported comparators assessed. The remaining 48% (n = 16), although providing information regarding the CRC screening strategies evaluated, did not state the reasons for their choice [22,27,29,30,32–36,38,44–46,48,49,51].

Time horizon adopted in each study was indicated in approximately 90% of the studies (n = 29) [20–31,34–36,38–46,48–52]. Nevertheless, only 48% (n = 16) provided a reason for the choice made [20,21,23,24,26–31,36,40,44,50,51]. Similarly, despite being reported in all studies, discount rate selection was not justified in more than half of included publications (n = 20, 61%) [22,27–30,32,33,35–40,43–49].

In a similar fashion, most studies (n = 18; 55%) did not provide justification for the economic model chosen [23–26,28,31,34,35,38,43–49,51,52]. In addition, although mentioning the modelling approach selected, the study from Hassan et al. did not provide information regarding the model structure [33].

An important proportion of the included studies did not adequately report information regarding the methods used to identify and synthesize effectiveness data (45%, n = 15) or to estimate resource use and costs (55%, n = 18) included in the simulations [22,27,29,30,33,35,38–40,42,44–47,49]. In addition, study parameters were not adequately reported in 36% of the studies (n = 12), as information regarding either probability distributions or reasons for distributions was mainly lacking [24,27,32,34,38,40,42–44,47,49,51]. Despite this, all the studies except three [21,46,49] adequately discussed the effects of sampling uncertainty on the estimated incremental cost and effectiveness parameters.

## Discussion

Economic evaluations play an important role in informing health policy decision making. In this report, we systematically reviewed and critically appraised the available economic evidence in the field of CRC screening. We observed a wide heterogeneity across studies in terms of characteristics of CRC screening strategies (i.e. periodicity, screening starting age, follow-up strategies for positive results etc.) and main assumptions used in the simulations. Therefore, it was not possible to draw conclusions regarding which strategy should be preferred for CRC screening at population level. In addition, several other factors should be considered that prevented us to combine results from different studies and identify a single strategy to be preferred over the others. Namely, CRC incidence varies between countries [1], thus, for instance the same CRC screening strategy may be associated with a higher number of cancer cases (and thus higher healthcare expenditure) in a population with higher CRC incidence in comparison to a population with a lower CRC incidence. Another factor to be considered is the differences in the structure of the healthcare system across countries. Studies from European countries (i.e. France, Germany, Netherlands, Ireland, England, Sweden) were conducted using the perspective of the national health system which provides, in all indicated countries except Ireland, a universal health coverage to their population [56]. Conversely, in the United States, health coverage is provided through a combination of private health insurance and public health coverage (e.g., Medicare, Medicaid) [57]. These differences in coverage translate into differences in the level of healthcare provision, the access to healthcare services and may even influence the adherence to screening programs. Indeed, out of pocket costs and limited financial resources have been significantly associated to lack of adherence to CRC screening [58]. These factors may thus influence the estimation of the effectiveness of CRC screening programs, affecting in turn the conclusions of the economic studies conducted. It should also be noted that both CRC screening test cost as well as healthcare services costs are largely different across countries, thus making it difficult to compare economic results obtained in studies conducted in different settings [20–52]. In addition, willingness to pay thresholds vary widely across countries [20–52]. In this sense, although a certain CRC screening approach might be considered cost-effective in a certain setting, this conclusion may not be extrapolated to a different setting, due to the differences in willingness to pay and the underlying health spending preferences.

Despite these differences, most of the studies suggested that implementing population-level CRC screening programs would be an efficient allocation of resources, as all assessed strategies were compared at least once with no screening and were deemed to be either cost-effective or cost-saving compared to it [20,21,23–25,27,28,30–43,45–52] These conclusions are in line with previous reports, evaluating the cost-effectiveness of CRC strategies in the United States (US) alone (Patel et al.) and in high-income countries (Ran et al.), respectively [59,60]. Similarly to what we observed, both studies found that most common CRC screening strategies were cost-effective compared to no screening [59,60]. Patel et al. also reported disagreement, across studies conducted in the US, as to which strategy was the most cost-effective [59]. In a similar fashion, Ran et al. highlighted the existence of a certain discrepancy among studies from different regions, which were mainly due to methodological differences and model assumptions [60]. Thus, in line with our conclusions, authors were not able to identify a unique optimal CRC screening strategy among those commonly used across countries [60].

Besides attempting to identify the most cost-effective CRC screening test, for its use in the general population, several studies included in the present review have tried to establish which should be the optimal starting age of screening [20,23,27,28,30,31,34,36,37,40,47,48,50,52]. Indeed, the optimal age for CRC screening is still debated and it has recently been advocated that starting age for CRC screening should be lowered to 45 years, as it would yield a better benefit/risk balance as compared to starting at 50 years [61]. Nevertheless, dropping the starting age to 45 years might be challenging due to increased resource use [61]. Before implementing this change, an analysis of whether the increased costs are offset by the health benefits provided and by potential savings in future expenditure due to avoided cancer cases, is needed. In the present review, only one study investigated the efficiency of lowering the screening starting age to 45 years [52]. The study, which was conducted in Canada and compared gFOBT, FIT and no screening, concluded that FIT every year between 45 and 80 years would be the more efficient alternative [52]. Further studies are needed to confirm these findings and evaluating the potential benefit of this strategy in other settings.

However, it must be noted that although age has been shown to have great value in predicting risk for CRC and appears to be useful for establishing a screening policy, age alone is not as effective in predicting risk when applied to individual patients [54]. Some authors claim that adherence to screening may probably be the most important factor to be considered when implementing a screening program [54].

This consideration is in line with the results of the bibliometric analysis performed in the present study which suggests that both the effectiveness of the chosen technique and individuals’ adherence to screening may influence the efficiency of CRC screening programs. Screening test sensitivity and adherence to screening, as well as screening test costs were frequently identified as main influential parameters on study results. This is not surprising if we consider that test performance and adherence to screening may vary depending on the test used for CRC screening [62]. For instance, colonoscopy is regarded as the gold standard screening, due to its high sensitivity and specificity [10]. Nevertheless, it has several drawbacks including the invasiveness of the procedure, the requirement of bowel preparation, as well as, a risk of bowel perforation [62]. These limitations contribute to low screening uptake and adherence to colonoscopy screening [62]. Conversely, less invasive techniques like FIT have lower sensitivity but have been associated with higher participation rates [62,63]. Thus, effectiveness and adherence to screening should be carefully analyzed when evaluating the convenience of implementing a CRC screening program.

In regard to the CRC screening strategies evaluated, the bibliometric analysis indicated that there is still limited evidence investigating the cost-effectiveness of new, less invasive screening tests such as CTC, fDNA, MT-sDNA, pDNA and mSEPT9. This might be partly explained by the limited availability of clinical evidence regarding the effectiveness of these techniques, thus complicating the development of robust economic analyses [10].

As for the reporting quality of selected studies, we observed that despite most of the studies showed an overall good adherence to CHEERS recommendations, there is still room for improvement. For instance, a relatively high percentage of studies (39%) did not provide enough information regarding the setting in which the decision as to which CRC screening strategy to adopt had to be made. In a similar fashion, although most of the studies reported the perspective of the analysis, almost a third of the studies, did not relate it to the costs being evaluated. In addition, perspective was not reported in 12% of the studies. Thus, in those studies, it was not possible to establish whether the resource use and associated costs included in the model were consistent with the objective of the analysis. Selection of time horizon and discount rate were also inadequately reported, with less than 50% of the studies justifying their selection.

According to the recommendations of the International Society for Pharmacoeconomics and Outcomes Research (ISPOR), systematic literature reviews should be conducted to ensure an adequate selection of data for input parameters to be included in decision-analytic models [55]. Thus, a description of the approach used to identify main input data of the analysis should accompany the model [55]. When assessing compliance to CHEERS recommendations regarding this aspect, we observed that a great proportion of included studies did not specify how the selection of both effectiveness and resource use data had been carried out. Thus, it was not possible to establish whether relevant information might have been missed during analysis design.

Overall, these results indicate that efforts should be made to improve reporting of several items (i.e. study settings, methods used for identification and selection of effectiveness and resource use data, study perspective and discount rate) which are considered necessary to establish the adequateness and robustness of cost-effectiveness analyses.

Our study is not free of limitations. Namely, results regarding main parameters affecting cost-effectiveness results, should be interpreted with caution. The degree to which each of the identified parameters impact studies’ results may vary depending on the main characteristics and design of the analyses conducted. As such, the heterogeneity observed between studies, may limit the generalization of our conclusions. Despite this, since numerous studies employed a similar modelling technique (i.e. Markov model) and perspective (health-care payer perspective), we believe the analysis performed may provide a good approximation and may be of use for future research, as it highlights main source of uncertainty that should be addressed both during analysis design and when interpreting cost-effectiveness results. In addition, it should be noted that despite conducting a comprehensive review of main databases of peer reviewed literature, we did not conduct a search of the grey literature, thus our conclusions may be partly affected by publication bias. It must also be acknowledged that although the inclusion in the scope of the review of new screening modalities has provided useful information regarding the status of the economic evidence currently available, it has also contributed to the heterogeneity of the study, thus limiting our ability to draw an overall conclusion regarding the preferred CRC strategy.

Finally, an intrinsic limitation connected to the use of CHEERS statement, as well as to other checklist to evaluate reporting quality of a study, is that the results of the quality assessment may vary depending on the assessor. To minimize this potential bias, all studies were evaluated by the same researcher.

## Conclusions

Our results indicate that implementing a CRC screening program at the population level, may provide good value for money compared to no screening. Further investigation is needed to assess the cost-effectiveness of more recently developed, less-invasive CRC screening tests, and to establish which of the available strategies should be preferred over the others. The assessment of reporting quality of available economic evidence suggests that efforts should be made to improve the reporting of relevant information regarding the context in which resource allocation decisions need to be made (i.e. relevant aspects of the study setting, study perspective and discount rate) as well as the methods used for the selection of input data.

## Supporting information

S1 Text Review protocol. (DOCX)

S2 Text Search strategies. (DOCX)

S3 Text Bibliometric analysis. (DOCX)

S4 Text Full data extraction. (DOCX)

S1 Table Main characteristics of selected studies. (DOCX)

S1 Fig Adherence to CHEERS recommendations. (TIF)

S1 File PRISMA checklist. (DOC)
