# Supplementary material for: Economic evaluations of screening strategies for the early detection of colorectal cancer in the average-risk population: A systematic literature review
Source: PLoS One. 2019 Dec 31;14(12):e0227251. doi: 10.1371/journal.pone.0227251 (PMC6938313; doi:10.1371/journal.pone.0227251)
Supplement: S1 Text — (DOCX) [file pone.0227251.s001.docx]

# Review protocol

## Rationale and objective

Colorectal cancer (CRC) is amongst the most common cancer worldwide [1] It is associated with high morbidity and mortality, accounting for approximately 1.8 million new cases and 880 792 deaths globally, in 2018 [1]. CRC screening in asymptomatic individuals has shown its ability to reduce both the incidence and mortality of CRC, allowing its identification at an early stage [2,3]. Based on these promising results, several countries worldwide have implemented screening programs to promote early detection of CRC [4]. Nevertheless, there is still debate regarding which screening strategy should be preferred among the ones available [5]. The choice of the screening strategy should be based both on the effectiveness of the test and on the economic consequences of implementing its use at population-level. Thus, the availability of high-quality economic evidence is essential to inform decision-making and ensure efficient resources allocation. Evidence regarding the cost-effectiveness of different CRC screening modalities has been previously reviewed and critically appraised in a work by Jeong et al that summarized economic evaluations published in English up to 2012[6]. Nevertheless, following the approval, in the latest years, of new CRC screening test, such as multi-target stool DNA test, methylated septin 9 assay etc., it seems necessary to provide an update of the economic evidence available in the field [7]. To the best of our knowledge, no report has been published which provide an update and appraise the economic evidence accumulated in the field worldwide. Based on this, we aim to systematically review and evaluate the reporting quality of full economic evaluations examining the cost-effectiveness of CRC screening tests, in the average risk population, published from January 2012 up to November 2018.

## Methodology

A systematic literature review will be carried out in in accordance with the Preferred Reporting Items for Systematic Reviews and Meta-Analyses (PRISMA) guidelines [8].

The aim of the review is to identify full economic evaluations (cost-effectiveness and cost-utility analyses) regarding the implementation of population-based CRC screening program.

### Bibliographic sources:

The following international databases will be searched for literature in English and Spanish:

- MEDLINE;
- EMBASE;
- Cost-Effectiveness Analysis (CEA) registry;
- National Health Service Economic Evaluation Database (NHS EED);
- Database of Abstracts of Reviews of Effects (DARE);
- EconLit;
- Health Technology Assessment (HTA) database.

In addition, the references of key articles will be revised to identify potentially relevant publications.

### Search strategies

A specific search will be used, depending on the characteristics of the database. Search strategies developed for each database are shown in the following tables

Table 1. Search strategy to be used for MEDLINE and EMBASE

| # | Search terms |
| --- | --- |
| 1 | ((colorectal or colon$ or rectum or rectal) adj2 (cancer$ or tumour$ or tumor$ or neoplasm$ or carcinoma$ or adenoma$ or polyp$)).ti,ab. |
| 2 | Colonoscopy/ |
| 3 | colonoscop$.ti,ab. |
| 4 | sigmoidoscop$.ti,ab. |
| 5 | exp Mass Screening/ |
| 6 | exp Population Surveillance/ |
| 7 | Diagnostic tests, routine/ |
| 8 | screen$.ti,ab. |
| 9 | 2 or 3 or 4 |
| 10 | 5 or 6 or 7 or 8 |
| 11 | 1 and 9 and 10 |
| 12 | limit 11 to yr="1999 -Current" |
| 13 | Economics/ or exp "Costs and Cost Analysis"/ or Economics, Dental/ or exp Economics, Hospital/ or exp Economics, Medical/ or Economics, Nursing/ or Economics, Pharmaceutical/ or Budgets/ or exp Models, Economic/ or Markov Chains/ or Monte Carlo Method/ or Decision Trees/ |
| 14 | (Economic* or cost or costs or costly or costing or costed or price or prices or pricing or pharmacoeconomic$ or pharmaco economic$ or budget*).ti,ab. |
| 15 | ((monte adj carlo) or markov or (decision adj2 (tree$ or analys$))).ti,ab. |
| 16 | Quality-Adjusted Life Years/ |
| 17 | (quality adjusted life or qaly*).ti,ab. |
| 18 | (disability adjusted life or daly).ti,ab. |
| 19 | (value adj2 (money or monetary)).ti,ab. |
| 20 | 13 or 14 or 15 or 16 or 17 or 18 or 19 |
| 21 | 12 and 20 |

Table 2. Search strategy to be used for NHS EED, DARE and HTA

| # | Searches |
| --- | --- |
| 1 | Colorectal cancer screening |
| 2 | Colon cancer screening |
| 3 | FOBT |
| 4 | Stool test |
| 5 | Colonoscopy |
| 6 | Colonography |
| 7 | Narrow-band imaging |
| 8 | Capsule endoscopy |
| 9 | Sigmoidoscopy |
| 10 | 1 or 2 or 3 or 4 or 5 or 6 or 7 or 8 or 9 |

Table 3. Search strategy to be used for EconLit

| # | Searches |
| --- | --- |
| 1 | Colorectal cancer screening |
| 2 | Colon cancer screening |
| 3 | FOBT |
| 4 | Stool test |
| 5 | Colonoscopy |
| 6 | Colonography |
| 7 | Narrow-band imaging |
| 8 | Capsule endoscopy |
| 9 | Sigmoidoscopy |
| 10 | 1 or 2 or 3 or 4 or 5 or 6 or 7 or 8 or 9 |

Table 4. Search strategy to be used for CEA Registry:

| # | Searches |
| --- | --- |
| 1 | Colorectal cancer screening |

### Time limits

The literature review will be limited to articles published in the last 6 years (from January 1, 2012; to November 22, 2018).

### Selection criteria

Only full economic evaluations (cost-effectiveness and cost-utility analysis) comparing costs and clinical benefits of CRC strategies, for the early detection of CRC in average-risk population, will be included in the review.

Studies will have to fulfill the following criteria:

- Population: average-risk population
- Intervention: CRC screening
- Comparator: alternative CRC screening strategy or no screening
- Outcome: cost per quality-adjusted life-year (QALY); or cost per life-year gained/saved (LYG/LYS)
- Language: English or Spanish.
- Publication date: between January 1, 2012 and November 22, 2018.

The following studies will be excluded:

- cost studies;
- disease burden studies;
- studies that do not match the research question;
- letters to the editor,
- editorials and clinical studies.

Abstracts and congress communications as well as narrative and systematic literature reviews will also be excluded as they may not provide sufficient details to allow for individual studies evaluation.

### Selection of studies

The selection of the retrieved publications will be done initially through the analysis of titles and abstracts to discard duplicates and non-relevant information (the one that is not related to the objective of the search). Potentially relevant publication will undergo full-text review, and inclusion and exclusion criteria will be applied for the final selection of key articles.

The selection will be carried out by two independent researchers. The selection discrepancies will be resolved by consensus.

### Data extraction

The following information will be extracted from selected publications:

- first author and year;
- country;
- study perspective;
- time horizon and cycles;
- population;
- interventions and comparators;
- modelling approach;
- effectiveness data sources;
- outcome measures;
- costs;
- cost data sources;
- year of costing;
- inflation adjustment;
- discount rate;
- reported results;
- sensitivity analysis;
- key variables influencing results;
- reported limitations;
- model validation;
- conclusions.

### Assessment of quality reporting

Reporting bias and overall reporting quality will be assessed using the Consolidated Health Economic Evaluation Reporting Standards (CHEERS) statement. The CHEERS statement consists of a 24-items checklist, covering six main categories: title and abstract, introduction, methods, results, discussion, source of funding, and conflicts of interest [10]. Reporting of each item will be assessed indicating “adequately reported” if recommendations are fully met, “inadequate reporting” if they are only partially fulfilled, “not reported” if they are not fulfilled and “not applicable” if reporting of the item is not required in the analyzed study. Overall adherence to CHEERS statement will be calculated for4 each study, as the percentage of adequately reported items.

### Bibliometric analysis

A bibliometric analysis of selected studies will be carried out to identify:

- most frequently assessed CRC screening techniques
- input parameters most frequently reported to influence results across studies.

Following extraction of data regarding CRC screening techniques evaluated, and influential parameters identified in the studies, data will be analyzed using VOSviewer (v. 1.6.5), a software tool for creating maps based on network data and for visualizing and exploring these maps [11]. A visualization map will be generated to visualize occurrence and co-occurrence of CRC screening strategies evaluated across studies [11]. Similarly, a visualization map will be generated to analyze occurrence and co-occurrence of parameters influencing the results [11].

# References

1. World Health Organization. Colorectal cancer Source: Globocan 2018 Number of new cases in 2018, both sexes, all ages [Internet]. 2018 [cited 30 May 2019]. Available at: http://gco.iarc.fr/today/data/factsheets/cancers/10_8_9-Colorectum-fact-sheet.pdf

2. Wilkins T, McMechan D, Talukder A. Colorectal Cancer Screening and Prevention. Am Fam Physician. 2018;97: 658–665.

3. Garborg K, Holme O, Loberg M, Kalager M, Adami HO, Bretthauer M. Current status of screening for colorectal cancer. Ann Oncol. 2013;24: 1963–1972.

4. Navarro M, Nicolas A, Ferrandez A, Lanas A. Colorectal cancer population screening programs worldwide in 2016: An update. World J Gastroenterol. 2017;23: 3632–3642.

5. Bénard F, Barkun AN, Martel M, von Renteln D. Systematic review of colorectal cancer screening guidelines for average-risk adults: Summarizing the current global recommendations. World J Gastroenterol. 2018;24: 124–138.

6. Jeong KE, Cairns JA. Review of economic evidence in the prevention and early detection of colorectal cancer. Health Econ Rev. 2013;3: 20.

7. Issa IA, Noureddine M. Colorectal cancer screening: An updated review of the available options. World J Gastroenterol. 2017;23: 5086.

8. Moher D, Liberati A, Tetzlaff J, Altman DG. Preferred Reporting Items for Systematic Reviews and Meta-Analyses: The PRISMA Statement. PLoS Med. 2009;6: e1000097.
