# Supplementary material for: Economic evaluations of screening strategies for the early detection of colorectal cancer in the average-risk population: A systematic literature review
Source: PLoS One. 2019 Dec 31;14(12):e0227251. doi: 10.1371/journal.pone.0227251 (PMC6938313; doi:10.1371/journal.pone.0227251)
Supplement: S2 Text — (DOCX) [file pone.0227251.s002.docx]

**S1 Text:** **Search strategies**

**Table A. Search strategy used for Ovid MEDLINE(R) ALL and EMBASE (2012 to 22/11/2018):**

| # | Searches | Results |
| --- | --- | --- |
| 1 | ((colorectal or colon$ or rectum or rectal) adj2 (cancer$ or tumour$ or tumor$ or neoplasm$ or carcinoma$ or adenoma$ or polyp$)).ti,ab. | 436173 |
| 2 | Colonoscopy/ | 94508 |
| 3 | colonoscop$.ti,ab. | 78087 |
| 4 | sigmoidoscop$.ti,ab. | 10761 |
| 5 | exp Mass Screening/ | 333742 |
| 6 | exp Population Surveillance/ | 266457 |
| 7 | Diagnostic tests, routine/ | 84703 |
| 8 | screen$.ti,ab. | 1567421 |
| 9 | 2 or 3 or 4 | 120882 |
| 10 | 5 or 6 or 7 or 8 | 2007654 |
| 11 | 1 and 9 and 10 | 21068 |
| 12 | limit 11 to yr="1999 -Current" | 10832 |
| 13 | Economics/ or exp "Costs and Cost Analysis"/ or Economics, Dental/ or exp Economics, Hospital/ or exp Economics, Medical/ or Economics, Nursing/ or Economics, Pharmaceutical/ or Budgets/ or exp Models, Economic/ or Markov Chains/ or Monte Carlo Method/ or Decision Trees/ | 1314111 |
| 14 | (Economic* or cost or costs or costly or costing or costed or price or prices or pricing or pharmacoeconomic$ or pharmaco economic$ or budget*).ti,ab. | 1621697 |
| 15 | ((monte adj carlo) or markov or (decision adj2 (tree$ or analys$))).ti,ab. | 146625 |
| 16 | Quality-Adjusted Life Years/ | 32855 |
| 17 | (quality adjusted life or qaly*).ti,ab. | 33060 |
| 18 | (disability adjusted life or daly).ti,ab. | 6836 |
| 19 | (value adj2 (money or monetary)).ti,ab. | 4937 |
| 20 | 13 or 14 or 15 or 16 or 17 or 18 or 19 | 2486840 |
| 21 | 12 and 20 | 1767:  1317 (EMBASE)  450 (Ovid Medline) |

**Table B. Search strategy used for NHS EED, DARE, HTA (2012 to 22/11/2018)**

| # | Searches | Results |
| --- | --- | --- |
| 1 | Colorectal cancer screening |  |
| 2 | Colon cancer screening |  |
| 3 | FOBT |  |
| 4 | Stool test |  |
| 5 | Colonoscopy |  |
| 6 | Colonography |  |
| 7 | Narrow-band imaging |  |
| 8 | Capsule endoscopy |  |
| 9 | Sigmoidoscopy |  |
| 10 | 1 or 2 or 3 or 4 or 5 or 6 or 7 or 8 or 9 | 193 |

**Table C. Search strategy used for EconLit (2012 to 22/11/2018)**

| # | Searches | Results |
| --- | --- | --- |
| 1 | Colorectal cancer screening |  |
| 2 | Colon cancer screening |  |
| 3 | FOBT |  |
| 4 | Stool test |  |
| 5 | Colonoscopy |  |
| 6 | Colonography |  |
| 7 | Narrow-band imaging |  |
| 8 | Capsule endoscopy |  |
| 9 | Sigmoidoscopy |  |
| 10 | 1 or 2 or 3 or 4 or 5 or 6 or 7 or 8 or 9 | 20 |

**Table D. Search strategy used for CEA Registry (2012 to 22/11/2018)**:

| # | Searches | Results |
| --- | --- | --- |
| 1 | Colorectal cancer screening | 13 |
