# Supplementary material for: Economic evaluations of screening strategies for the early detection of colorectal cancer in the average-risk population: A systematic literature review
Source: PLoS One. 2019 Dec 31;14(12):e0227251. doi: 10.1371/journal.pone.0227251 (PMC6938313; doi:10.1371/journal.pone.0227251)
Supplement: S3 Text — (DOCX) [file pone.0227251.s003.docx]

**S2 Text: Bibliometric analysis**

**Table A. Grouping and occurrence of CRC screening strategies evaluated in selected studies.**

| **CRC screening technique** | **N.º of occurences** |
| --- | --- |
| No screening | 28 |
| FIT | 23 |
| COL | 22 |
| gFOBT | 14 |
| FS | 10 |
| FIT + FS | 6 |
| FS + gFOBT | 5 |
| COL + FIT | 4 |
| CTC | 4 |
| gFOBT + Other:   - gFOBT + COL*; gFOBT + CTC + COL*; gFOBT + CTC/ COL* - gFOBT + hrfq - gFOBT+ FIT | 3 |
| fDNA | 2 |
| mSETP9 | 2 |
| COL + Other:   - COL + Aspirin - COL + Calcium*; COL + Aspirin* + Calcium; COL+Aspirin* | 2 |
| FIT + pDNA | 1 |
| pDNA | 1 |
| Double contrast enema | 1 |
| FIT/COL | 1 |
| gFOBT/COL | 1 |
| FS + Aspirin | 1 |
| MRC | 1 |
| MT-sDNA | 1 |

*These techniques were evaluated in the same publication, thus, since they were grouped under the same label, they were only considered once by VOSviewer.

**Table B. Grouping and occurrence of key influential parameters identifies across studies**

| **Influential parameter** | **N.º of occurences** |
| --- | --- |
| Screening test cost | 19 |
| Adherence to screening | 12 |
| Screening test sensitivity | 9 |
| Cost of CRC treatment | 9 |
| Screening test specificity | 6 |
| Screening uptake | 5 |
| Discount rate | 6 |
| Screening starting age:   - Screening starting age - Age of screening | 3  2  1 |
| Time horizon | 2 |
| Differential screening utilization | 1 |
| Risk of CRC after screening | 2 |
| CRC incidence:   - CRC incidence - Annual CRC incidence | 2  1  1 |
| CRC prevalence | 1 |
| Disease progression | 1 |
| Follow-up of positive results | 1 |
| Inclusion of extracolonic findings | 1 |
| Screening threshold | 1 |
| Testing interval | 1 |
| Annual transition probabilities between health states | 1 |
| COL demand | 1 |
| CRC prevention | 1 |
| Initial prevalence of adenomas | 1 |
