# Supplementary material for: Economic evaluations of screening strategies for the early detection of colorectal cancer in the average-risk population: A systematic literature review
Source: PLoS One. 2019 Dec 31;14(12):e0227251. doi: 10.1371/journal.pone.0227251 (PMC6938313; doi:10.1371/journal.pone.0227251)
Supplement: S4 Text — (DOCX) [file pone.0227251.s004.docx]

# S4 Text Data extraction

Table 1. Data extraction of Lew et al. 2018.(1).

| Item | Data |
| --- | --- |
| Author | Lew et al. |
| Year | 2018 |
| Country | Australia |
| Perspective | Health services perspective |
| Time horizon and cycles | 20-90 years or death; with cycles of 1-year duration |
| Population | 20-75 years for screening purpose |
| Intervention(s) | iFOBT 2y; iFOBT 1y; pDNA2y;fDNA5y;COL10y; SIG10y; CTC10y; SIG@60;SIG@55_iFOBT2y@60To74;COL@50_iFOBT2y@52To74; FOBT2y1SIG@50;iFOBT2y1SIG@54_64_74; iFOBT2y1plasmaDNA. |
| Comparator (s) | No screening |
| Modelling approach | Individual-level model (microsimulation model): Policy 1- bowel.  It includes both the adenoma-carcinoma pathway and the serrated pathway in colorectal cancer development, assuming 15% of colorectal cancers are attributable to the serrated pathway. |
| Effectiveness data sources | Literature review and Australian reimbursement data. |
| Outcomes measures | Cost-effectiveness ratio compared to no screening.  Incremental cost-effectiveness ratio for each dominating strategy vs. the next most effective dominating strategy. |
| Costs | Invitation letter (except iFOBT), Screening test, GP consultation for abnormal screening result or referral letter, colonoscopy (for patients with positivity of iFOBT, pDNA or fDNA) CRC treatment. |
| Cost data sources | Literature review and Australian reimbursement data. |
| year of costing | Varies depending on cost data source |
| Inflation adjustment | Not clearly stated |
| Discount rate | Discount rate of 5% starting from age 40 years as per a predicate Medical Services Advisory Committee (MSAC) evaluation of the National Cervical Screening Program. |
| Reported results | When compared with no screening, all strategies were estimated to be associated with a ICER close to or lower than the indicative WTP threshold in Australia of A$50,000/ LYS in all three scenarios of adherence. iFOBT was the most cost-effective strategy in all three scenarios. |
| Sensitivity analysis | One-way sensitivity analysis.  Supplementary analysis was performed to assess the impact of the simulation stop age on the predicted health and cost effectiveness by repeated simulations for all screening strategies. |
| Key variables influencing results | No variable was shown to significantly influence results. |
| Reported limitations | Analyses for fDNA, pDNA and CTC were considered exploratory since modelling was based on cross-sectional observational data on test characteristics, given no longitudinal data on longer term outcomes were available.  Influential parameters including screening test costs, screening participation and screening test performance for alternative modalities to iFOBT were based on assumptions, by necessity. Quality of life was not incorporated in the analysis. |
| Model validation | It was adapted from an existing colorectal cancer natural history model, the Adenoma and Serrated pathway to Colorectal CAncer (ASCCA) model and was extensively re-calibrated jointly to the original natural history data and the Australian setting. Detailed calibration and validation results for the Australian implementation have been performed (reference is provided). |
| Conclusions | 2-yearly screening using iFOBT in people aged 50 to 74 years is one of the most effective options for bowel cancer screening in Australia and is also cost-effective. |

Table 2. Data extraction of Van der Meulen et al. 2018 (2)

| Item | Data |
| --- | --- |
| Author | van der Meulen et al. |
| Year | 2018 |
| Country | Netherlands |
| Perspective | Third-party payer |
| Time horizon and cycles | Lifetime time horizon |
| Population | Age distribution of the Dutch population aged 25-85 years  Screening naïve members of the general population aged 50–75 years and living in the regions of Amsterdam and Rotterdam (population included in the COCOS trial) |
| Intervention(s) | CT colonography; colonoscopy |
| Comparator (s) | No screening |
| Modelling approach | Microsimulation Model. (Adenoma-carcinoma pathway) |
| Effectiveness data sources | The Colonoscopy or Colonography for Screening, or COCOS, trial |
| Outcomes measures | Costs and quality-adjusted life-years (QALYs) gained for all screening strategies compared with no screening. |
| Costs | Screening costs, costs for non-responder, diagnostic costs inside screening program, costs of complications after colonoscopy, treatment costs. |
| Cost data sources | The Colonoscopy or Colonography for Screening, or COCOS, trial. |
| year of costing | 2012 (original year of costing from data sources not reported). |
| Inflation adjustment | Inflation adjusted to 2012 euros by using the Dutch Consumer Price Index. |
| Discount rate | 3% for costs and benefits. In the Sensitivity analysis1.5% for QALYs and 4% for costs according to Dutch guidelines. |
| Reported results | Screening every 10 years:  With a 100% participation rate, screening once every 10 years from age 50 to 70 years resulted in a higher mortality reduction and more QALYs gained with colonoscopy than with CT colonography (106 vs 81 QALYs gained, 24% lower). As a result, colonoscopy dominated CT colonography screening. With observed participation, colonography screening resulted in a higher mortality reduction, more QALYs gained (29 vs 22 QALYs gained, 34% higher) but still higher total costs.  Comparison of CT Colonography and Colonoscopy  With observed participation rates, colonoscopy screening with one or two lifetime screenings was less costly than and just as effective as the same CT colonography strategies. However, with more lifetime screening, CT colonography screening dominated the colonoscopy strategies. The first CT colonography strategy on the efficient frontier had a screening age of 55–70 years, an interval of 5 years, and an ICER of €3162 per QALY gained. The most cost-effective CT colonography strategy with an ICER below the €20 000 threshold was CT colonography triennially from age 45 to 80 years (ICER €14 709 per QALY gained). |
| Sensitivity analysis | One-way sensitivity analysis and scenario analysis |
| Key variables influencing results | Inclusion of extracolonic findings;  Participation rates;  Screening costs. |
| Reported limitations | Lacking data on participation to subsequent screening rounds. Exposure to ionizing and extracolonic findings at CT colonography were not included.  Authors did not model distinct pathways for traditional and sessile serrated adenomas and/or polyps. |
| Model validation | Not reported |
| Conclusions | Because of the higher participation rates, CT colonography screening for colorectal cancer is more cost-effective than colonoscopy screening. The implementation of CT colonography screening requires previous satisfactory resolution to the question as to how best to deal with extracolonic findings. |

Table 3. Data extraction of Melnitchouk et al. 2018 (3)

| Item | Data |
| --- | --- |
| Author | Melnitchouk et al. |
| Year | 2018 |
| Country | Ukraine |
| Perspective | Ministry of Health of Ukraine |
| Time horizon and cycles | Lifetime time horizon Cycle length: 1 year |
| Population | 50 years with screening up to 75 years |
| Intervention(s) | FOBT every year followed by colonoscopy if positive;  Flexible sigmoidoscopy every 5 years with FOBT every year, followed by colonoscopy if positive;  Colonoscopy every 10 years. |
| Comparator (s) | No screening |
| Modelling approach | Markov model. (From polyp to cancer pathway) |
| Effectiveness data sources | Data from the published literature to obtain estimates the prevalence of the polyps and age-specific transition probabilities. Stage-specific CRC mortality with treatment estimated using US data from the Surveillance, Epidemiology and End Results (SEER) database.  National Cancer Registry of Ukraine database to estimate current Ukrainian CRC incidence, mortality, and treatment adherence. |
| Outcomes measures | Lifetime costs, effects and Incremental cost-effectiveness ratio (ICER). |
| Costs | Direct treatment and screening costs. |
| Cost data sources | Screening costs were based on the manufacturer’s price for FOBT and on prices charged by private medical institutions in Ukraine. Treatment costs were based on chemotherapy third party prices that the Ministry of Health of Ukraine pays for medications, as documented on the Ministry’s website. Surgical and radiation therapy costs were obtained from private clinics and modified them to reflect what would be reasonable in the public sector based on expert opinion from personnel at the National Cancer Institute of Ukraine. |
| year of costing | 2012 (original year of costing not reported). |
| Inflation adjustment | all costs were translated to 2012 US dollars. |
| Discount rate | Future costs and effectiveness discounted at an annual rate of 3%. |
| Reported results | The base-case lifetime cost-effectiveness analysis showed that all three screening strategies were cost saving compared to no screening, and among the three strategies, colonoscopy every 10 years was the dominant strategy compared to no screening with standard adherence to treatment.  When decreased adherence to treatment was modeled, colonoscopy every 10 years was the most cost-effective strategy with an incremental cost-effectiveness ratio of $843 per QALY compared with no screening.  Sensitivity analysis results: The optimal screening scenario changes from colonoscopy every 10 years to sigmoidoscopy with FOBT when the costs of colonoscopy exceed the threshold value of $236 and the probability of compliance with colonoscopy falls below the threshold value of 0.66. |
| Sensitivity analysis | One-way, Two-way and three-way sensitivity analysis. |
| Key variables influencing results | cost of colonoscopy;  decreased compliance with colonoscopy; |
| Reported limitations | The costs were calculated from the payer perspective instead of societal perspective.  The model did not incorporate the potentially high cost of establishing a national screening program in Ukraine, including the costs of addressing adherence, public health campaigns to change attitudes or other methods at the provider level to increase adherence. |
| Model validation | Comparison between model simulation of CRC incidence and observed incidences from the National Cancer Registry of Ukraine in 2013–2014. |
| Conclusions | Colonoscopy every 10 years is superior to the other screening modalities evaluated in this  study. This knowledge can be used to concentrate efforts on developing a national screening program in Ukraine. |

Table 4. Data extraction of Greuter et al. 2017 (4)

| Item | Data |
| --- | --- |
| Author | Greuter et al. |
| Year | 2017 |
| Country | Netherlands |
| Perspective | Health care payer |
| Time horizon and cycles | Lifetime time horizon |
| Population | Asymptomatic persons aged 55 to 75 years without a prior CRC diagnosis |
| Intervention(s) | FIT screening with and without colonoscopy surveillance |
| Comparator (s) | No screening or surveillance |
| Modelling approach | Microsimulation using the ASCCA (Adenoma and Serrated pathway to Colorectal CAncer) model |
| Effectiveness data sources | Dutch CRC screening program and published literature. |
| Outcomes measures | CRC burden, colonoscopy demand, life-years, and total lifetime costs. |
| Costs | FIT, colonoscopy and CRC treatment |
| Cost data sources | Dutch CRC screening program and published literature. |
| year of costing | Costs are reported in the tables in 2015 U.S. dollars using the purchasing power parity for that year |
| Inflation adjustment | Costs were converted to 2016 euros using the consumer price index for that year |
| Discount rate | Costs and effects were discounted at 3% annually |
| Reported results | FIT screening without surveillance reduced CRC mortality by 50.4% compared with no screening or surveillance. Adding surveillance to FIT screening reduced mortality by an additional 1.7% to 52.1% but increased lifetime colonoscopy demand by 62% at an additional cost of €68 000, for an increase of 0.9 life-year.  Extending the surveillance intervals to 5 years reduced CRC mortality by 51.8% and increased colonoscopy demand by 42.7% compared with FIT screening without surveillance. In an incremental analysis, incremental cost-effectiveness ratios (ICERs) for screening plus surveillance exceeded the Dutch willingness-to-pay threshold of €36 602 per life-year gained. |
| Sensitivity analysis | 1-way sensitivity analysis |
| Key variables influencing results | Colorectal lesion prevalence; colonoscopy costs; colorectal lesion incidence |
| Reported limitations | Limited data on FIT performance and background CRC risk in the surveillance population. |
| Model validation | Not reported |
| Conclusions | Adding surveillance to FIT screening is not cost-effective based on the Dutch ICER threshold and substantially increases colonoscopy demand. Extending surveillance intervals to 5 years would decrease colonoscopy demand without substantial loss of effectiveness. |

Table 5 Data extraction of Aronsson et al. 2017 (5)

| Item | Data |
| --- | --- |
| Author | Aronsson et al. |
| Year | 2017 |
| Country | Sweden |
| Perspective | Healthcare payer perspective |
| Time horizon and cycles | Lifetime time horizon with 1-year cycles |
| Population | hypothetical population of Swedish 60-year-olds |
| Intervention(s) | Colonoscopy once, colonoscopy every 10 years, FIT twice, FIT biennially and no screening |
| Comparator (s) | No screening |
| Modelling approach | Markov decision analysis model |
| Effectiveness data sources | Sensitivity of colonoscopy was retrieved from a meta-analysis reported  by Telford and colleagues, and the corresponding values for FIT were from de Wijkersloot et al. (published literature) |
| Outcomes measures | Cost saving compared with not screening, avoided colorectal cancer, added life-years, QALYs and incremental cost per QALY. |
| Costs | Invitation to screening, material for FIT x 2, laboratory cost of FIT x 2, colonoscopy and polypectomy |
| Cost data sources | Assumption, published literature and Board of health and welfare |
| year of costing | 2016 (adjusted) |
| Inflation adjustment | Not reported |
| Discount rate | Costs and effects were discounted at 3% annually |
| Reported results | it was estimated that screening once with colonoscopy yielded 49 more quality-adjusted life-years (QALYs) and a cost saving of €64 800 compared with no screening. Similarly, screening twice with FIT gave 26 more QALYs and a cost saving of €17 600. When the colonoscopic screening was repeated every tenth year, 7 additional QALYs were gained at a cost of €189 400 compared with a single colonoscopy. The additional gain with biennial FIT screening was 25 QALYs at a cost of €154 300 compared with two FITs. |
| Sensitivity analysis | Two- way sensitivity analyses |
| Key variables influencing results | Discount rate; starting age; disease progression. |
| Reported limitations | the difficulty in including all the effects of  screening and only colonoscopy and FIT were compared with no screening |
| Model validation | Not reported |
| Conclusions | All strategies that included screening were cost-effective compared with no screening. Repeated and single screening with colonoscopy were more cost-effective than FIT when lifelong effects and costs were considered. A single screening colonoscopy yielded the lowest cost per QALY, whereas screening every tenth year gained additional QALYs at a reasonable cost. |

Table 6. Data extraction of Goede et al. 2017 (6)

| Item | Data |
| --- | --- |
| Author | Goede et al. |
| Year | 2017 |
| Country | Canada |
| Perspective | Third party health-care payer |
| Time horizon and cycles | Lifetime time horizon |
| Population | A cohort of 40-year-old screening participants at average risk of CRC |
| Intervention(s) | gFOBT and FIT (FIT 50, FIT 75, FIT 100, FIT 150 and FIT 200). After positive test result individuals were referred for diagnostic colonoscopy. |
| Comparator (s) | Not screening |
| Modelling approach | MISCAN-Colon microsimulation model |
| Effectiveness data sources | Three large gFOBT trials. (Published literature)  FIT positivity rates and detection rates of adenomas and CRC observed in the first screening round of two Dutch randomized trials.  The test characteristics of colonoscopy were based on a systematic review of polyp miss rates in tandem colonoscopy studies |
| Outcomes measures | QALYs, costs and number of colonoscopies, compared to no screening. |
| Costs | Screening, per event of: gFOBT, FIT, GP visit after positive stool test, colonoscopy (no polypectomy), colonoscopy (polypectomy), bleeding (complication), perforation (complication)  Treatment per person year of CRC care: stage I, stage II, stage III, stage IV. |
| Cost data sources | Colon Cancer Check program, Ontario Ministry of Health and Long-term Care and published literature. |
| year of costing | 2013 Canadian dollars |
| Inflation adjustment | Not reported |
| Discount rate | Costs and QALYs were discounted by 3% per year |
| Reported results | Compared to no screening, biennial gFOBT screening between age 50±74 years provided 20 QALYs at a cost of CAN$200,900 per 1,000 participants and required 17 colonoscopies per 1,000 participants per year. FIT screening was more effective and less costly. For the same level of colonoscopy requirement, biennial FIT between age 50±74 years provided 11 extra QALYs gained while saving CAN$333,300 per 1000 participants, compared to gFOBT. Without restrictions in colonoscopy capacity, FIT every year between age 45±80 years was the most cost-effective strategy providing 27 extra QALYs gained per 1000 participants, while saving CAN$448,300. |
| Sensitivity analysis | One-way sensitivity analysis |
| Key variables influencing results | Colonoscopy and treatment costs |
| Reported limitations | Uncertainty in assumptions used in the model and several assumptions were therefore based on international data.  Assumed perfect adherence to screening, follow-up and surveillance and not explicitly model distinct pathways for traditional and sessile serrated adenomas. |
| Model validation | Yes, MISCAN -colon model is a model used in previous publications |
| Conclusions | Compared to gFOBT screening, switching to FIT at a high cut-off level could increase the health benefits of a CRC screening program without considerably increasing colonoscopy demand. |

Table 7. Data extraction of Coldman et al. 2017 (7).

| Item | Data |
| --- | --- |
| Author | Coldman et al. |
| Year | 2017 |
| Country | Canada |
| Perspective | Third party payer |
| Time horizon and cycles | Lifetime time horizon |
| Population | Cohort aged 45 years; screening between ages 50–74 years |
| Intervention(s) | FIT |
| Comparator (s) | No screening |
| Modelling approach | OncoSim Model |
| Effectiveness data sources | Systematic review published, Cancer Risk Management Model, and individual published articles |
| Outcomes measures | ICER, cost/QALY |
| Costs | Not reported |
| Cost data sources | Ontario Health Insurance Plan and Ontario Case Costing Initiative |
| year of costing | Not reported |
| Inflation adjustment | 2014 Canadian Dollars (CAD$) |
| Discount rate | 3% |
| Reported results | Reducing the FIT threshold reduced both projected CRC incidence and mortality for all parameter sets, although cost impacts and cost-effectiveness varied compared to no screening. Biennial FIT was projected to be cost-effective at all thresholds considered with a maximum of CAD$ 5400 per QALY over the seven parameter sets and a maximum of CAD$ 6800 per QALY for a one level change in the eight threshold levels considered. Demand for colonoscopy varied strongly with FIT threshold and was greatest for the lowest threshold (50 ng/ml) but the magnitude varied across the 7 parameter sets. |
| Sensitivity analysis | Scenario analysis (threshold variation) |
| Key variables influencing results | Colonoscopy demand |
| Reported limitations | The present study did not attempt to adjust for differences in the age and sex composition and location of studies used to inform TPR’s so that true differences in TPR performance are likely over estimated since variation in the composition of the population will have been included as uncertainty in the TPR‘s. |
| Model validation | Yes, OncoSim-CRC has been previously described, validated, and used to compare different modalities and options for CRC screening. |
| Conclusions | Compared to no screening, all thresholds of FIT examined were predicted to be cost-effective in the prevention and management of CRC. Threshold choice strongly influences predicted demand placed upon colonoscopy resources. |

Table 8. Data extraction of Murphy et al. 2017 (9)

| Item | Data |
| --- | --- |
| Author | Murphy et al. |
| Year | 2017 |
| Country | England |
| Perspective | Health-care system (NHS/BCSP) |
| Time horizon and cycles | 60-100 years or death; cycles of 1-year duration |
| Population | Individuals at age 60 years; screened from age 60-74 years |
| Intervention(s) | Faecal immunochemical test for haemoglobin (FIT) |
| Comparator (s) | Guaiac faecal occult blood test (gFOBT) |
| Modelling approach | Cohort-based Markov state transition model |
| Effectiveness data sources | FIT sensitivity and specificity relative to gFOBT were estimated using the detection rates from the BCSP FIT pilot study. |
| Outcomes measures | Incremental cost of FIT versus gFOBT, life years and quality-adjusted life years (QALYs) |
| Costs | CRC management costs, screening costs, colonoscopy-related costs |
| Cost data sources | Screening and colonoscopy costs were taken from NHS or BCSP sources.  Costs of CRC management were taken from a model-based evaluation of CRC services by Pilgrim et al. No cost was assigned to death. |
| year of costing | Not specified, costs adjusted to 2015/2016 prices |
| Inflation adjustment | All costs were adjusted, where necessary, to 2015/2016 prices using the Health Service Cost Index. |
| Discount rate | 3.5% per year from age 60 years to the end of the time horizon at age 100 years for costs and quality of life outcomes |
| Reported results | FIT is cost-effective compared with gFOBT at all thresholds, resulting in cost savings and quality-adjusted life years (QALYs) gained over a lifetime time horizon.  FIT was cost-saving (p<0.001) and resulted in QALY gains of 0.014 (95% CI 0.012 to 0.017) at the base case threshold of 180 μg Hb/g faeces. Greater health gains and cost savings were achieved as the FIT threshold was decreased due to savings in cancer management costs.  However, at lower thresholds, FIT was also associated with more colonoscopies (increasing from 32 additional colonoscopies per 1000 people invited for screening for FIT 180 μg Hb/g faeces to 421 additional colonoscopies per 1000 people invited for screening for FIT 20 μg Hb/g faeces over a 40-year time horizon). Parameter uncertainty had limited impact on the conclusions. |
| Sensitivity analysis | Probabilistic sensitivity analysis and one-way sensitivity analysis |
| Key variables influencing results | Cancer management costs  FIT sensitivity |
| Reported limitations | The sensitivity and specificity of gFOBT and FIT were not directly observed in the BCSP pilot study population, so they estimated the FIT parameters using screening data for FIT relative to gFOBT.  They modelled a cohort starting screening at age 60 years and continuing until death. Further modelling would be required to take into account multiple cohorts starting FIT screening at different ages. |
| Model validation | The model structure was developed based on a previously validated model for the NHS BCSP. |
| Conclusions | Results suggest that FIT is highly cost-effective compared with gFOBT at all thresholds for a cohort aged 60 years at first screen in England. Further modelling is needed to estimate economic outcomes for screening across all age cohorts simultaneously. |

Table 9. Data extraction of Greuter et al. 2016 (10)

| Item | Data |
| --- | --- |
| Author | Greuter et al. |
| Year | 2016 |
| Country | The Netherlands |
| Perspective | Healthcare perspective |
| Time horizon and cycles | 20-90 years or death (lifetime time horizon); each cycle has a length of 1 year |
| Population | Healthy individuals aged 20 years. |
| Intervention(s) | CTC, MRC, colonoscopy and FIT. Twelve strategies were compared.  CTC 55 and 65 yrs;  CTC 55, 65 and 75 yrs;  CTC 50, 60, 70 and 80 yrs;  CTC 55, 60, 65, 70 and 75 yrs  MRC 55 and 65 yrs;  MRC 55, 65 and 75 yrs;  MRC 50, 60, 70 and 80 yrs;  MRC 55, 60, 65, 70 and 75 yrs  Colonoscopy 55 and 65 yrs;  Colonoscopy 55, 65 and 75 yrs:  Colonoscopy 50, 60, 70 and 80 yrs.  FIT screening 55-75 yrs (every two yrs). |
| Comparator (s) | No screening, three rounds of colonoscopy screening |
| Modelling approach | Markov model (ASCCA model) Adenoma-carcinoma pathway |
| Effectiveness data sources | Test characteristics were based on meta-analyses on CTC, screening trials and a meta-analysis on MRC1and a systematic review on colonoscopy.  Assumptions: FIT sensitivities were estimated via calibration of the model-predicted FIT positivity and detection rates for advanced adenomas and CRC to figures reported for a Dutch FIT screening trial.  Published literature. |
| Outcomes measures | Total cost per individual, LYG, ICER |
| Costs | CTC costs (invitational costs, positive scan, negative scan, complications after CTC), MRC costs (invitational costs, costs per scan, complications after MRC), colonoscopy costs (invitational costs, without polypectomy, with polypectomy, complications after colonoscopy), FIT costs (testkit, organization, analysis) and CRC treatment costs (stage I, stage II, stage III, stage IV) |
| Cost data sources | Published literature, Health council of Netherlands, Nederlandse Zorgautoriteit |
| year of costing | 2014 |
| Inflation adjustment | Consumer price index for year 2014 |
| Discount rate | 3% |
| Reported results | All imaging-based strategies were cost-effective compared with no screening. FIT screening was the dominant screening strategy, leading to most LYG and highest cost-savings. Compared with three rounds of colonoscopy screening, CTC with five rounds was found to be cost-effective in an incremental analysis of imaging strategies. |
| Sensitivity analysis | One-way sensitivity analysis |
| Key variables influencing results | Participation rates; tests costs;  Positivity rate for small and large adenomas |
| Reported limitations | A limitation of the present study is that the participation rate of CTC was derived from a study using limited bowel preparation, whereas the test characteristics of CTC were derived from studies using full bowel preparation.  In addition, costs of CRC treatment were based on a study reporting treatment costs in Ireland in 2008. |
| Model validation | Not reported |
| Conclusions | imaging-based CRC screening is cost-effective compared with no screening. CTC screening is favoured over MRC screening owing to the lower costs per test. Furthermore, an incremental analysis of imaging strategies showed that CTC screening with five rounds is a cost-effective alternative for three rounds of colonoscopy screening. Compared with FIT screening, imaging-based screening is unlikely to be ever cost-effective. Participation is an important driver of effectiveness and cost estimates |

Table 10. Data extraction of Ladabaum et al. 2016(11).

| Item | Data |
| --- | --- |
| Author | Ladabaum et al. |
| Year | 2016 |
| Country | US |
| Perspective | Third-party payer (Medicare and commercial  insurance payments) |
| Time horizon and cycles | 50-100 years or death;1-year |
| Population | Persons 50 years old |
| Intervention(s) | MT-sDNA every 3 years, FIT every 2 years, FIT yearly |
| Comparator (s) | No screening, |
| Modelling approach | Markov model |
| Effectiveness data sources | Fit, and MT-sDNA sensitivity from a Multitarget stool DNA testing for colorectal-cancer screening study and Colonoscopy sensitivity from a systematic review and individual study |
| Outcomes measures | CRC cases, CRC stage, CRC deaths, QALYs/person, cost/person, ICER, colonoscopies/person and fecal tests/person |
| Costs | FIT, MT-sDNA, colonoscopy, colonoscopy with lesion removal, major hemorrhage after colonoscopy, perforation after colonoscopy. |
| Cost data sources | Centers for Medicare and Medicaid Services and published literature |
| year of costing | 2015 |
| Inflation adjustment | The medical component of the consumer price index |
| Discount rate | 3% |
| Reported results | With optimal adherence, yearly FIT and colonoscopy every 10 years were dominant than MT-sDNA every 3 years. Compared with successful organized FIT programs, the patient support program for the MT-sDNA test would need 68% of subjects to participate consistently and 32% to participate intermittently every 3 years, or the MT-sDNA test would need to cost 60% less than in the base case, for the MT-sDNA test to be preferred over FIT at a threshold of $100,000 per quality-adjusted life-year (QALY) gained. performing the MT-sDNA test every 3 years would cost less than $100,000 per QALY gained if the MT-sDNA test achieved a participation rate more than 1.7-fold that of FIT. The results were robust in sensitivity analyses. |
| Sensitivity analysis | One- way and multiway sensitivity analyses |
| Key variables influencing results | Sensitivity of strategy, annual cost of strategy, testing interval; |
| Reported limitations | Not reported |
| Model validation | Yes, the model it’s an adaptation of a validation model |
| Conclusions | In conclusion, at comparable levels of screening participation, a program of screening for colorectal neoplasia with MT-sDNA is likely to be dominated by programs based on FIT or colonoscopy. MT-sDNA may be a cost-effective alternative if it can achieve patient participation rates that are high enough compared with those of FIT that paying for its higher test cost can be justified. |

Table 11. Data extraction of Wong et al. 2016(12).

| Item | Data |
| --- | --- |
| Author | Wong et al. |
| Year | 2016 |
| Country | China |
| Perspective | Not reported |
| Time horizon and cycles | 50-70 years (20 years time horizon) |
| Population | Asymptomatic subjects aged 50 years |
| Intervention(s) | flexible sigmoidoscopy (FS) 5 yearly;  colonoscopy 10 yearly;  FS for each woman at 50- and 55-year old followed by colonoscopy at 60- and70-year old;  FS for each woman at 50-, 55-, 60-, and 65-year old followed by colonoscopy at 70-year old;  FS for each woman at 50-, 55-,60-, 65-, and 70-year old. |
| Comparator (s) | No screening |
| Modelling approach | Markov model |
| Effectiveness data sources | Published literature |
| Outcomes measures | Total number CRC cases, total loss of cancer related life years, cases of CRC prevented, proportion of CRC case prevented, life-years saved, number of procedures, number of complications, costs, care of CRC, total costs per life-years saved and ICER switching form screening method 1 to 2. |
| Costs | FS, colonoscopy, consultation fee, bleeding, polypectomy, perforation, treatment for the stage I or II of CRC, treatment for the stage II and treatment for the stage IV. |
| Cost data sources | Hong Kong Government Gazette |
| year of costing | Not reported |
| Inflation adjustment | Not reported |
| Discount rate | 3% |
| Reported results | strategy 2 could save the largest number of life-years (4226 vs 2268 to 3841 by other strategies). When compared with no screening, strategy 5 had the lowest ICER (US$42,515), followed by strategy 3 (US$43,517), strategy 2 (US$43,739), strategy 4 (US$47,710), and strategy 1 (US$56,510). Strategy 2 leads to the highest number of bleeding and perforations and required a prohibitive number of colonoscopy procedures. |
| Sensitivity analysis | 1-way sensitivity analyses and probabilistic analysis |
| Key variables influencing results | Reduced FS compliance rate, increasing specificity of FS and cost of colonoscopy |
| Reported limitations | Firstly, the participants are self-referred and might not be representative of the general public. Also, although we used actual data in the Markov models, some variables made reference to published studies due to the relatively short period (5 years) of follow-up of the screening cohort. Furthermore, more conservative screening strategies may refer only 3% to 7% of patients with advanced distal neoplasia at index FS for colonoscopy follow-up,44 and even lower proportions in subsequent FS screening tests. Another example is that a certain proportion of cancer polyps might only be treated with endoscopy but not surgery, and this may bear implications on the cost estimates. Similar arguments exist for the compliance rates for FS and Also, since our modeling and cost estimates were based on CRC instead of advanced neoplasia (AN), the distribution of AN in subgroups might be more applicable in future modeling studies which compared strategies with AN as an outcome variable. Lastly, this study has not evaluated the cost-effectiveness of some hybrid strategies. |
| Model validation | Not reported |
| Conclusions | this study highlighted the higher cost-effectiveness of implementing tailored CRC screening programs based  on age and gender. These findings could inform physicians and policy-makers in triaging eligible subjects for risk-based screening—especially in countries with limited colonoscopic resources. |

Table 12. Data extraction of Espinola et al. 2016(13).

| Item | Data |
| --- | --- |
| Author | Espinola et al. |
| Year | 2016 |
| Country | Argentina |
| Perspective | Third party payer |
| Time horizon and cycles | 50years;1 year |
| Population | General population of average risk (persons asymptomatic and with no family or personal history related to RCC). |
| Intervention(s) | screening program, with annual screening program using iFOBT, with LOC screening program every 10 years |
| Comparator (s) | No screening |
| Modelling approach | Markov model |
| Effectiveness data sources | COLO sensitivity was estimated from a Clinical trial and clinical guidelines.  iFOBT form different published studies |
| Outcomes measures | QALYs and ICER |
| Costs | specialist consultation, immunochemical test, colonoscopy, polypectomy, biopsy, pathological anatomy surgical piece, cancer treatment stage I, II, III and IV annual follow-ups |
| Cost data sources | institutions for the management of social security and prepaid resources and the SSS (Super intendencia de Servicios de Salud) |
| year of costing | 2014 |
| Inflation adjustment | Not reported |
| Discount rate | 3% |
| Reported results | The most cost-effective strategy consisted annual FIT, in comparison no intervention and colonoscopy every 10 years. The incremental cost effectiveness ratio (ICER) of FIT versus no intervention was of 980.5 pesos per QALY. |
| Sensitivity analysis | 1-way sensitivity analysis |
| Key variables influencing results | Average costs of diagnosis and treatment of CRC |
| Reported limitations | Not reported |
| Model validation | Not reported |
| Conclusions | We confirmed that screening for CRC is a cost-effective intervention. Results support the widespread use of screening for CRC using annual FIT, which proves to be highly cost effective in the setting considered. |

Table 13. Data extraction of Hassan et al. 2015 a (14).

| Item | Data |
| --- | --- |
| Author | Hassan et al. |
| Year | 2015 |
| Country | United States |
| Perspective | Not stated |
| Time horizon and cycles | Not reported |
| Population | 50-80 years |
| Intervention(s) | Low-ADR, Average-ADR and High-ADR, overall colonoscopy screening |
| Comparator (s) | No screening |
| Modelling approach | Markov model |
| Effectiveness data sources | Randomized controlled trial |
| Outcomes measures | CRC cases, CRC prevented, CRC prevention rate, CRC deaths, CRC death prevention rate, life-years gained, screening cost, care for CRC (cost/person), total cost/person and ICER vs no screening |
| Costs | Polypectomy, follow-up, endoscopy, related complications, CRC treatment at the different stages and annual wage for an American endoscopist |
| Cost data sources | Medicare, Bureau of Labor Statistics |
| year of costing | Not reported |
| Inflation adjustment | Not reported |
| Discount rate | 3% |
| Reported results | Screening colonoscopy performed by endoscopists with low adenoma detection rates resulted in a 7% absolute reduction in the long-term colorectal cancer incidence prevention rate as compared to the same procedure performed by those with an average adenoma detection rate (70% vs. 77%). This difference increased to 21% when comparing endoscopists with an average with those with a high adenoma detection rate. When projected on the US population, this reduced efficacy resulted in an additional 1728 and 16,123 colorectal cancer cases and the loss of $117 million and $906 million per year in the two scenarios, respectively. These estimates were sensitive to the risk of post-colonoscopy interval colorectal cancer.  ICER vs no screening, $ per life-year saved – 4,424 |
| Sensitivity analysis | Two-way sensitivity analysis |
| Key variables influencing results | Risk of post-colonoscopy interval colorectal cancer |
| Reported limitations | The main limitation of our analysis is represented by the lack of studies showing a higher long-term CRC prevention rate when screening colonoscopy is performed by endoscopists with high or average ADR. |
| Model validation | Not reported |
| Conclusions | A substantial reduction in long-term colorectal cancer prevention rate may be expected when screening colonoscopy is performed by endoscopists with a suboptimal adenoma detection rate. A substantial saving may be expected when implementing policies to improve endoscopist adenoma detection rate.  When subgrouping endoscopists with different ADR, a 7%–21% reduction in long-term CRC prevention rate may be estimated, resulting in a substantial loss of life-years and economic resources |

Table 14. Data extraction of Wong et al. 2015(16).

| Item | Data |
| --- | --- |
| Author | Wong et al. |
| Year | 2015 |
| Country | China |
| Perspective | Health service provider |
| Time horizon and cycles | 25 years or death; 1-year |
| Population | Persons from 50 years old Hong Kong population |
| Intervention(s) | Annual g-FOBT; annual i-FOBT; g-FOBT q2y; biennial i-FOBT; colonoscopy every 10 years |
| Comparator (s) | No screening |
| Modelling approach | Markov model |
| Effectiveness data sources | Sensitivities and specificities associated with G-FOBT and I-FOBT were based  on the results of two local Hong Kong studies, while assumptions were used for colonoscopy |
| Outcomes measures | Lifetime medical costs per person for all screening strategies, Lys, QALYs, ICER |
| Costs | cost of cancer care, screening test, outpatient follow-up, screening complications |
| Cost data sources | Hong Kong study, government gazette, cost-effectiveness analysis studies (published literature) |
| year of costing | 2009 |
| Inflation adjustment | Local costs evaluated in Hong Kong dollar (year 2009 values) were converted to US dollar at the pegged exchange rate of USD 1 = HKD 7.8. |
| Discount rate | 3.5% |
| Reported results | In base-case scenario, the non-dominated strategies were annual and biennial I-FOBT. Compared with no screening, the ICER presented $20,542/LYs and $3155/QALYs gained for annual I-FOBT, and $19,838/LYs gained and $2976/QALYs gained for biennial I-FOBT. The optimal screening strategy was annual I-FOBT that attained the highest ICER at the threshold of $50,000 per LYs or QALYs gained. |
| Sensitivity analysis | Deterministic (univariate and multivariate) and probabilistic sensitivity analysis (PrSA) were performed |
| Key variables influencing results | The most sensitive collection of clinical parameters was the natural history parameters representing the annual transition probabilities between health states. Decreased specificity of I-FOBT was associated with an increased in ICER for annual I-FOBT compared with biennial I-FOBT. |
| Reported limitations | Several limitations with respect to the model assumptions should be noted. First, results were primarily simulated by Markov modeling. Authors assumed that the disease progression and cost spending were the same in the tumour locations of colon and rectum. Second, the utility data was measured by cross-sectional study rather than randomized controlled trial with sufficient follow-up periods, which involves the consideration of time-dependent utility data in the short and long term. |
| Model validation | Yes (external validation) |
| Conclusions | The Markov model informed the health policymakers that I-FOBT every year may be the most effective and cost-effective CRC screening strategy among recommended screening strategies, depending on the willingness to- pay of mass screening for Chinese population |

Table 15. Data extraction of Sekiguchi et al. 2015(17).

| Item | Data |
| --- | --- |
| Author | Sekiguchi et al. |
| Year | 2016 |
| Country | Japan |
| Perspective | National health care payer |
| Time horizon and cycles | Lifetime ;1-year |
| Population | 40 years who were at an average risk of CRC |
| Intervention(s) | Annual FIT; colonoscopy every 10 years; FIT 40 + colonoscopy 50 |
| Comparator (s) | No screening |
| Modelling approach | Markov model |
| Effectiveness data sources | Published literature |
| Outcomes measures | ICER, cost per person, QALYs, CRC cases, colonoscopy procedures |
| Costs | FIT, colonoscopy, endoscopic resection of low-risk polyp, endoscopic resection of high-risk polyp, annual cost of CC management |
| Cost data sources | Japanese national reimbursement tables |
| year of costing | Not reported |
| Inflation adjustment | Not reported |
| Discount rate | 3% |
| Reported results | Among the three strategies, simple dominance of Strategy 3 over Strategy 1 was observed: Strategy 3 resulted in more QALYs and less cost than Strategy 1. Compared with Strategies 1 and 3, Strategy 2 yielded more QALYs, but involved greater cost. The ICERs per QALY gained for Strategy 2 against Strategies 1 and 3 were JPY 293 616 and JPY 781 342, respectively. |
| Sensitivity analysis | Scenario analyses and probabilistic sensitivity analysis |
| Key variables influencing results | With regard to the optimal age for population-wide TCS in Strategy 3, TCS at 45 years was the most cost-effective under the condition of the upper limit of WTP being JPY 5-6 million, according to the scenario analyses in this study. Considering that it is necessary to set the age for population-wide TCS as a range rather than one specific age to achieve a higher uptake rate, it appears that TCS within the age range 45–55 years would be acceptable from the perspective of cost-effectiveness on the basis of the study results. |
| Reported limitations | This study had several limitations. First, the natural history model of CRC in this study was based on currently available Japanese data. Second, the values of model parameters set in the base case analysis could vary case by case in the real world. Third, indirect costs such as productivity loss cost due to CRC treatment were not considered in this study. |
| Model validation | Yes (comparing the lifetime cumulative risks for CRC incidence and mortality for the 40-year-old Japanese population estimated from the model of this study with those estimated from Japan’s Cancer Registry and Statistics) |
| Conclusions | The CRC screening strategies with more active use of TCS could be more cost-effective than the FIT-based screening strategy. The TCS-based screening strategy could be the most cost-effective; however, considering the safety and limited capacity of TCS resources in addition to cost effectiveness, the strategy of adding population-wide TCS for individuals in the age range 45-55 years to the FIT-based screening may be an optimal solution. |

Table 16. Data extraction of Hassan et al. 2015 b (18).

| Item | Data |
| --- | --- |
| Author | Hassan et al. |
| Year | 2015 |
| Country | United States |
| Perspective | Social perspective |
| Time horizon and cycles | Not reported |
| Population | 50 to 100 years old, who had never previously undergone colonoscopy, had no known personal history of inflammatory bowel disease, polyposis syndrome, or previous colon resection surgery, and had no family history of CRC (average risk of CRC) |
| Intervention(s) | SFV-colonoscopy and FUSE- colonoscopy |
| Comparator (s) | No screening |
| Modelling approach | Markov model |
| Effectiveness data sources | randomized reported per-patient adenoma detection rates, calculus |
| Outcomes measures | CRC prevented, CRC incident prevention, life-years saved, colonoscopies performed, therapeutic (with polypectomy), diagnostic (with polypectomy), cost of colonoscopy, cost of care for CRC, total cost, ICER |
| Costs | Screening provider cost, screening site of performance costs, median hourly income rate |
| Cost data sources | National Compensation Survey: Occupational Earnings in the United States, 2010 and assumption |
| year of costing | 2014 |
| Inflation adjustment | the medical component of the consumer price index for that year |
| Discount rate | 3% |
| Reported results | The significantly higher sensitivity of full spectrum endoscopy in detecting additional adenomas resulted in an increase in cancer prevention from 58% to 74%. This 14% increase led to an absolute reduction in the cost of cancer care from $90 million to $57 million. This cost savings was only minimally impacted by the higher cost of more frequent post polypectomy colonoscopy surveillance rates, leading to full spectrum endoscopy being associated with a savings of $145 per person. Thus, standard colonoscopy appeared to be “dominated” by the full spectrum endoscopy. |
| Sensitivity analysis | One- and two- way sensitivity analysis |
| Key variables influencing results | adherence to colonoscopy, annual incidence |
| Reported limitations | The primary limitation of our model is that the FUSE colonoscopy test characteristics were based only on one study. Secondly, cost-effective models do not represent clinical trials and are limited by the correctness of model inputs and assumptions. |
| Model validation | Not reported |
| Conclusions | FUSE colonoscopy appears to be a cost-effective, and potentially cost-saving option in CRC screening and surveillance. In particular, the higher associated costs of more frequent post-polypectomy colonoscopy surveillance are compensated by the significant overall reduction in CRC treatment costs. |

Table 17. Data extraction of Huang et al. 2014(19).

| Item | Data |
| --- | --- |
| Author | Huang et al. |
| Year | 2014 |
| Country | China |
| Perspective | third-party payer’s perspective |
| Time horizon and cycles | 35 years |
| Population | Average risk asymptomatic individuals 40 years old |
| Intervention(s) | FOBT initial screening instrument (with 4 different scenarios of follow-up) and FOBT+HRFQ as an initial screening instrument (with 4 different scenarios of follow-up) |
| Comparator (s) | No screening |
| Modelling approach | Markov model |
| Effectiveness data sources | Published literature evaluating the effectiveness of colorectal cancer screening |
| Outcomes measures | Direct costs, effectiveness measures (discount life years lost, life years saved, CRC accumulated cases, CRC deaths, CRC prevented) and ICER |
| Costs | Marketing, material and distribution and return of FOBT and FOBT+HRFQ, pathology, colonoscopy, polypectomy and treatment of CRC |
| Cost data sources | Published study comparing and evaluating screening programs for colorectal cancer in urban communities in China; Bureau of National Health Insurance |
| year of costing | 2008 |
| Inflation adjustment | Not reported |
| Discount rate | 3% |
| Reported results | The study revealed that a combined use of FOBT and HRFQ is preferable in CRC screening programs as an initial screening instrument. Annual FOBT+HRFQ screening is recommended for those who have a negative initial result and those who have a positive result but have failed to continue to colonoscopy examination. Repeated colonoscopy (for those with a positive result in initial screening but a negative colonoscopy result) should be performed at a ten-year interval instead of one-year. Such a protocol would cost 7732 Yuan per life year saved, which is the most cost-effective option. |
| Sensitivity analysis | One-way and two-way sensitivity analyses were applied to assess the influence of those parameters on ICER. |
| Key variables influencing results | colonoscopy request compliance increased compared with that when coverage of initial screening increased, rising sensitivity of initial screening, rising specificity of initial screening and discount rate |
| Reported limitations | In this study, we only calculated direct costs. Indirect costs such as those associated with production loss due to attending screening and treatment services should be considered in future studies. |
| Model validation | Not reported |
| Conclusions | A combined use of FOBT and HRFQ is preferable in CRC screening programs as an initial screening instrument. Annual FOBT +HRFQ screening is recommended for those who have a negative initial result and those who have a positive result but have failed to comply with colonoscopy procedures. Repeated colonoscopy should be performed at a ten-year interval instead of one-year. |

Table 18. Data extraction of Ladabaum et al. 2014(20).

| Item | Data |
| --- | --- |
| Author | Ladabaum et al. |
| Year | 2014 |
| Country | Germany |
| Perspective | National health care payer |
| Time horizon and cycles | 100 years or death; 1-year |
| Population | persons at average risk for CRC from the ages of 50 to 75 years |
| Intervention(s) | COLO 60,70; mSEPT9-2well q2; FOBT; mSEPT9-3well q2; COLO 55,65; FOBT/COLO 60,70; mSEPT9-2well q1; mSEPT9-3well q1; FIT/COLO 60,70; FOBT/COLO 55,65, FIT and FIT/COLO 55,65 |
| Comparator (s) | No screening |
| Modelling approach | Markov model |
| Effectiveness data sources | Published literature |
| Outcomes measures | CRC cases, CRC deaths, QALYs, cost per person, ICER, colonoscopies required |
| Costs | SEPT9, FOBT, FIT, colonoscopy, colonoscopy with lesion removal, major hemorrhage after colonoscopy, perforation after colonoscopy, CRC care by stage |
| Cost data sources | literature review, the doctor’s fee scale and procedure reimbursement catalogue 2011 for office-based physicians with an EBM point value of € 0.035, and German Diagnostic Related Group (DRG) codes for hospitalizations |
| year of costing | 2011 |
| Inflation adjustment | Not reported |
| Discount rate | 3% |
| Reported results | FIT was more effective and less costly than mSEPT9 testing. FIT/COLO 55,65 cost €12200 per quality-adjusted life-years gained in comparison with FIT. mSEPT9-based screening was cost-effective in comparison with no screening but was dominant ed by other cost-saving strategies.  FIT was preferred in 49% and FIT/COLO 55,65 in 47% of iterations |
| Sensitivity analysis | One-way and probabilistic analysis |
| Key variables influencing results | Differential screening utilization and adherence |
| Reported limitations | This is a modeling study, and the results depend directly on the model inputs and assumptions. We have assumed conditional independence between repeated rounds of testing, which may not be true. No longitudinal data are available on the utilization of and adherence to current screening strategies over time in Germany. There are no data on these parameters for emerging blood-based biomarkers.  Our focus on specific comparisons and threshold analyses addresses the a priori aims of this study, but it does not imply that these strategies are preferred, especially in light of the uncertainties surrounding utilization and adherence. Finally, our CRC treatment cost inputs were not based on a cost-of-illness study, but instead on expert opinions and other sources |
| Model validation | Yes (Three validation exercises against data from the U.K. Flexible Sigmoidoscopy Screening Trial, the SCORE trial, and the PLCO (Prostate, Lung, Colorectal and Ovarian) Cancer Screening Trial.) |
| Conclusions | The currently recommended and covered CRC screening strategies in Germany, including hybrid strategies of fecal-based testing and colonoscopy, are likely to be cost-saving. The place of emerging blood-based biomarkers such as mSEPT9 among screening options in Germany will depend not only on their test performance characteristics and cost, but also on their utilization and longitudinal adherence over time compared with the accepted alternatives. |

Table 19. Data extraction of Lejeune et al. 2014(21).

| Item | Data |
| --- | --- |
| Author | Lejeune et al. |
| Year | 2014 |
| Country | France |
| Perspective | funding sources |
| Time horizon and cycles | 85 years or death; |
| Population | 50-74 years |
| Intervention(s) | Magstream 1-stool sample – 20 ng/mL; FOB-Gold 1-stool sample – 176 ng/mL; G-FOBT; FOB-Gold 2-stool samples – 352 ng/mL; FOB-Gold 2-stool samples – 293 ng/mL; Magstream 2-stool samples – 20 ng/mL; FOB-Gold 2-stool samples – 234 ng/mL; OC-Sensor 1-stool sample – 150 ng/mL; FOB-Gold 2-stool samples – 205 ng/mL; FOB-Gold 2-stool samples – 176 ng/mL; OC-Sensor 2-stool samples – 300 ng/mL; OC-Sensor 2-stool samples – 250 ng/mL; OC-Sensor 2-stool samples – 200 ng/mL; OC-Sensor 2-stool samples – 175 ng/mL; OC-Sensor 2-stool samples – 150 ng/mL |
| Comparator (s) | Guaiac test |
| Modelling approach | Markov model |
| Effectiveness data sources | Published literature |
| Outcomes measures | Cost of the screening programme, number of life-years lost, ICER |
| Costs | Screening campaign organization cost, information cost, test distribution cost, processing cost, and cost of CRC or advanced adenoma treatment and follow-up |
| Cost data sources | Previously published cost-effectiveness analysis, micro-costing method based on data provided by the coordination and centralized analysis centers of the IGOR Burgundy study |
| year of costing | 2011 |
| Inflation adjustment | Not reported |
| Discount rate | 3% |
| Reported results | Immunochemical tests were efficient strategies compared with guaiac faecal occult blood test. When all 15 strategies were compared with each other, only five of them remained efficient: the one and two-stool sample Magstream, the one- and two-stool sample FOB-Gold with the 176 ng/mL cut-off, and the two-stool sample OC-Sensor with the 150 ng/mL cut-off. |
| Sensitivity analysis | deterministic sensitivity analyses |
| Key variables influencing results | Test cost |
| Reported limitations | At first, we did not take into account the possible multiple adenomas that could result in a higher probability of having a positive test than individuals presenting a single lesion. Another limitation is the exclusion in the false positive rate of the presence of small and medium adenomas. We assumed a 100% colonoscopy sensitivity for colorectal lesions, and we did not take into account the possible false negative cases. we excluded from the analysis some indirect costs corresponding to short-term and long-term disabilities, and costs due to premature deaths from CRC. Complications due to colonoscopy were not taken into account in the model. Data concerning treatment cost only covered the first year after diagnosis. Finally, we made the choice to perform a cost-effectiveness analysis and not a cost utility based on the use of Quality-adjusted Life-Years (QALYs). |
| Model validation | yes |
| Conclusions | One-stool immunochemical testing can be considered a promising alternative to the guaiac faecal occult blood test for colorectal cancer mass screening in the general population |

Table 20. Data extraction of Hashimoto et al. 2014(22).

| Item | Data |
| --- | --- |
| Author | Hashimoto et al |
| Year | 2014 |
| Country | Japan |
| Perspective | Health care payer’s perspective. |
| Time horizon and cycles | 20 years; 1-year |
| Population | 40 years old |
| Intervention(s) | Strategy 1: FOBT-positive cases would be offered CTC, CTC positive would undergo OC  Strategy 2: FOBT-positive cases would be offered OC, only those who were reluctant to undergo OC would be offered CTC. |
| Comparator (s) | FOBT followed by OC |
| Modelling approach | Markov model |
| Effectiveness data sources | Previous Japanese and foreign studies and clinical experts |
| Outcomes measures | Cost, CRC death, expected life-year, QALY, ICER |
| Costs | CTC, OC, polypectomy (≤2 cm in diameter and ≥2 cm in diameter), malignant tumor resection (colon and rectum), postoperative chemotherapy, chemotherapy, follow-up, annual cost bay CRC stage |
| Cost data sources | National Health Insurance Drug Price Standard 2010, Drugs in Japan, Ethical Drugs 2012 and guidelines |
| year of costing | Not reported |
| Inflation adjustment | Not reported |
| Discount rate | 3% |
| Reported results | In the base-case (20-year) analysis, total cost was increased from Japanese yen (JPY) 65,614 million (strategy 1) to JPY 69,405 million (strategy 2) but was decreased to JPY 63,878 million (strategy 3). The total QALY increased from 28,156,046 QALYs (strategy 1) to 28,158,349 (strategy 2) and 28,159,058 QALYs (strategy 3). Therefore, the incremental cost-effectiveness ratio was JPY 1,646,000 per QALY gained for strategy 2 and strategy 3 was dominant against strategy 1, both of which were well below the Japanese threshold (JPY 5–6 million per QALY gained). |
| Sensitivity analysis | Scenario and probabilistic sensitivity analyses |
| Key variables influencing results | Uptake of FOBT, uptake of CTC, uptake of OC, time horizon, discount rate, and the cohort aged 50 years with a10-year time horizon. |
| Reported limitations | In this study, we constructed the model so that it worked adversely on introducing CTC where data were ambiguous, because colorectal cancer epidemiological data were not sufficient. We did not consider the adverse events that may occur with OC or CTC. |
| Model validation | Not reported |
| Conclusions | The cost-effectiveness of CTC for a colorectal cancer screening program in the working age population in Japan was as follows. The ICER was JPY1,646,000 per QALY gained for strategy 2 and strategy 3 was dominant against strategy1, both of which were well below the Japanese threshold (JPY5–6 million per QALY gained). These ICER values will be further improved with a longer time horizon than used in the basic analysis. Therefore, adding CTC into the current colorectal cancer screening program for the working age population seems to be a cost-effective option. |

Table 21. Data extraction of Dinh et al. 2013(23).

| Item | Data |
| --- | --- |
| Author | Dinh et al. |
| Year | 2013 |
| Country | United States |
| Perspective | Third party provider (Kaiser Permanente) |
| Time horizon and cycles | 30 years |
| Population | 50-75 years |
| Intervention(s) | Sigmoidoscopy; FIT; Annual FIT/ COLOx1; Concurrent FIT/ sigmoidoscopy; colonoscopy |
| Comparator (s) | No screening |
| Modelling approach | Archimedes model (simulation model) |
| Effectiveness data sources | COLO: literature review and a cost-saving analysis  FIT: published literature including a comparative evaluation and a comparison with total colonoscopy |
| Outcomes measures | CRC cases, CRC deaths, number of FITs needed, number of colonoscopies needed, cost per person, QALYs per person, incremental QALYs per person (compared with the strategies), incremental cost per QALY gained (compared with the strategies) |
| Costs | FIT, biopsy/polypectomy, colonoscopy, sigmoidoscopy and treatment for CRC by stages and phases |
| Cost data sources | Medicare reimbursement rate |
| year of costing | 2010 |
| Inflation adjustment | Costs were adjusted to 2010 using the medical care component of the Bureau of Labor Statistics Consumer Price Index |
| Discount rate | 3% |
| Reported results | Screening by annual FIT of patients 50–65 years old and then a single colonoscopy when they were 66 years old (FIT/COLOx1) reduced CRC incidence by 72% and gained 110 QALYs for every 1000 people during a period of 30 years, compared with no screening.  Compared with annual FIT, FIT/COLOx1 gained 1400 QALYs/100,000 persons at an incremental cost of $9700/QALY gained and required 55% fewer FITs. Compared with FIT/COLOx1, colonoscopy at 10-year intervals gained 500 QALYs/100,000 at an incremental cost of $35,100/QALY gained but required 37% more colonoscopies. |
| Sensitivity analysis | one-way sensitivity analyses |
| Key variables influencing results | discount rate, compliance to colonoscopy |
| Reported limitations | The model was principally built on U.S. data, which limit the potential applications of the model to other countries.  The present model contains a simplified description of treatment and diagnosis.  Several important risk factors are not included in the model, including smoking, use of HRT, and consumption of red meat.  The model relies heavily on CORI data obtained in 2007, which only provides information on polyp size and location, not polyp type (e.g. adenomatous versus benign). We assume that the characteristics (size, anatomical distribution) of benign polyps are similar to those of adenomas. This is the best assumption that we can make given the lack of data.  We have not assessed the effects of uncertainties in parameter estimations.  The current model only captures tubular and villous adenomas. It does not include sessile serrated adenomas. |
| Model validation | Yes, validated against a wide range of CRC studies including the National Polyp Study, Minnesota FOBT Screening Trial, Cancer Prevention Study II Nutrition Cohort,32 Women’s Health Initiative, the UK Flexible Sigmoidoscopy Trial, and the Veterans Affairs Cooperative Study Group. |
| Conclusions | A strategy of annual or biennial FIT, beginning when patients are 50 years old, with a single colonoscopy when they are 66 years old, delivers clinical and economic outcomes similar to those of CRC screening by single-modality strategies, with a favorable impact on resources demand. |

Table 22. Data extraction of Barouni et al. 2013(24).

| Item | Data |
| --- | --- |
| Author | Barouni et al. |
| Year | 2013 |
| Country | Iran |
| Perspective | Health insurance organization |
| Time horizon and cycles | Lifetime; 1-year |
| Population | Iranian of 50-year-old average-risk for colon cancer |
| Intervention(s) | Annual gFOBT; Annual iFOBT; COL q10y |
| Comparator (s) | No screening |
| Modelling approach | Markov model |
| Effectiveness data sources | Published literature and Surveillance, Epidemiology and End Results database. |
| Outcomes measures | QALY, ICER |
| Costs | Not reported |
| Cost data sources | Published literature |
| year of costing | Not reported |
| Inflation adjustment | Inflation adjustment to USD 2011 |
| Discount rate | 5% |
| Reported results | These strategies reduced the incidence of colorectal cancer by 39%, 60%, and 76% and mortality by 50%, 69%, and 78%, respectively, compared with no screening. These strategies generated ICER (incremental cost-effectiveness ratios) of $9067, $654, and $8700 per QALY (quality-adjusted life year), respectively. |
| Sensitivity analysis | One-way and probabilistic sensitivity analyses |
| Key variables influencing results | Sensitivity of the test to detect advanced adenoma, cost of the test, compliance with screening and cost of cancer care |
| Reported limitations | Model based economic evaluation depends on the data available in the medical literature, which is constantly evolving.  The natural history of colorectal cancer is based on assumptions regarding the progression from adenoma to carcinoma and transition time from a low- risk polyp to a malignant neoplasm. We did not include the possibility or regression of polyps.  Other limitations were related to incorporating the following untested assumptions: characteristics of test performance would remain constant on repeat testing, incidence of adenoma would be unaffected by screening and compliance with testing was random.  The model did not incorporate the costs or establishing the infrastructure to implement population- based screening for colorectal cancer.  Lost productivity costs, which are necessary to determine the societal perspective, were not incorporated. |
| Model validation | Not reported |
| Conclusions | Screening for colorectal cancer is cost-effective over conventional levels of WTP (Willingness to Pay). Annual high-sensitivity fecal occult blood testing, such as a fecal immunochemical test, or colonoscopy every 10 years offer the best value for the money in Iran. |

Table 23. Data extraction of Pence et al. 2013(25).

| Item | Data |
| --- | --- |
| Author | Pence et al. |
| Year | 2013 |
| Country | United States |
| Perspective | Not stated |
| Time horizon and cycles | 25 years; 1-year |
| Population | 50 years old with a colonoscopy compliance rate of 50% |
| Intervention(s) | COLO; Aspirin + COLO; Calcium +COLO; Aspirin + Calcium + COLO |
| Comparator (s) | No screening |
| Modelling approach | Markov model |
| Effectiveness data sources | Published literature (individual articles) |
| Outcomes measures | CRC cases, CRC prevented, CRC deaths, LYS, CFYS, Strategy cost, care for CRCs, total cost, ICER |
| Costs | Annual aspirin treatment, annual calcium treatment, colonoscopy, colonoscopy with polypectomy, complication costs (average cost of CRC treatment, colonoscopy complication and aspirin-related complication) |
| Cost data sources | CVS Pharmacy, Centers for Medicare & Medicaid Services, cost effectiveness study, published literature |
| year of costing | Not clearly stated for all costs, probably 2011 |
| Inflation adjustment | Not reported |
| Discount rate | 3% |
| Reported results | The ICER per LYS for colonoscopy alone dominated compared with no screening. Compared with colonoscopy alone, colonoscopies with aspirin (ICER $12,950/LYS) or calcium (ICER $13,041/LYS) were the next most cost-effective strategies. CERs per CFYS were $3,061 and $2,317 for aspirin and calcium, respectively, when added to colonoscopy |
| Sensitivity analysis | One- and two-way sensitivity analysis |
| Key variables influencing results | Initial prevalence of adenomas |
| Reported limitations | We did not include the indirect costs of CRCs in the analysis, nor did we include the suboptimal efficacy of colonoscopy screening for proximal CRC, In addition, we did not include as inputs to our model, the additional deaths prevented from cardiovascular disease, other cancers, preventive effects of calcium supplementation on fracture risk, and other competing causes of mortality that could also have been prevented by aspirin and calcium. The Another possible limitation to our analysis is the apparent discrepancy between the numbers of cancers in the no screening scenario, which are higher than Surveillance Epidemiology and End Results (SEER) data for lifetime probability of developing CRCs. |
| Model validation | Not reported |
| Conclusions | Low-dose aspirin or calcium supplementation may be beneficial when added to colonoscopy, for optimum CRC prevention, at small incremental costs.  Cost-effectiveness analyses suggest that aspirin and calcium in combination with colonoscopies are cost-effective for CRC prevention in average-risk populations |

Table 24. Data extraction of Goede et al. 2013(27).

| Item | Data |
| --- | --- |
| Author | Goede et al. |
| Year | 2013 |
| Country | The Netherlands |
| Perspective | Healthcare system |
| Time horizon and cycles | Lifetime time horizon |
| Population | 45-80 years |
| Intervention(s) | One-sample FIT; Two-sample FIT, at least one sample positive; Two-sample FIT, mean of both samples positive; Two-sample FIT, both samples positive |
| Comparator (s) | No screening |
| Modelling approach | Microsimulation model |
| Effectiveness data sources | first screening round of two Dutch randomized trials |
| Outcomes measures | LYG, costs, ICER |
| Costs | FIT costs (costs per invitation, costs per attendee), colonoscopy costs (without polypectomy, with polypectomy), costs complications after colonoscopy, treatments costs (initial treatment, continuous care, terminal care death CRC, terminal car death other) stage I, II, II, IV. |
| Cost data sources | Dutch cervical cancer screening programme, prices from the manufacturer, Dutch Health Care Authority, Dutch Health Care Insurance Board, internal 6 months study at the Erasmus MC |
| year of costing | Not clearly stated, probably 2005 |
| Inflation adjustment | Not reported |
| Discount rate | 3% |
| Reported results | Within an exemplary screening strategy, biennial FIT from the age of 55e75 years, one-sample FIT provided 76.0e97.0 life-years gained (LYG) per 1000 individuals, at a cost of V259 000e264 000 (range reflects different FIT cut-off levels). Two-sample FIT screening with at least one sample being positive provided 7.3e12.4 additional LYG compared with one-sample FIT at an extra cost of V50 000e59 000. However, when all screening intervals and age ranges were considered, intensifying screening with one-sample FIT provided equal or more LYG at lower costs compared with two-sample FIT. |
| Sensitivity analysis | Yes |
| Key variables influencing results | Cost difference between one-sample FIT over two-sample FIT |
| Reported limitations | First, we based our analysis on data from one screening round.  Second, we assumed the screening attendance rate to be independent of screening intensity and the number of FIT samples performed.  Third, we based our analyses on a screening-naive population.  Finally, we did not perform a probabilistic sensitivity analysis. |
| Model validation | Yes, they used a published model |
| Conclusions | If attendance to screening does not differ between strategies it is recommended to increase the number of screening rounds with one-sample FIT screening, before considering increasing the number of FIT samples provided per screening round. |

Table 25. Data extraction of Sharaf et al. 2013(28).

| Item | Data |
| --- | --- |
| Author | Sharaf et al. |
| Year | 2013 |
| Country | United States |
| Perspective | Perspective of an insurer such as Medicare |
| Time horizon and cycles | 50 years; 1-year |
| Population | 50-years, average risk persons |
| Intervention(s) | Annual FOBT, annual FIT, FS, COLO, FS/FIT, FS (once) |
| Comparator (s) | No screening |
| Modelling approach | Markov model |
| Effectiveness data sources | Published literature including cost-effectiveness analyses, systematics reviews and individual studies |
| Outcomes measures | CRC cases, CRC stage, fraction of all deaths attributable to CRC, deaths attributable to endoscopic complications, QALYs/person, cost/person, ICER, resources demand (colonoscopies per person, sigmoidoscopies per person, population in surveillance at age 65 years and population in surveillance at age 71 years) |
| Costs | FOBT, FIT, sigmoidoscopy, sigmoidoscopy with biopsy, colonoscopy, colonoscopy with lesion removal, major hemorrhage after sigmoidoscopy or colonoscopy and perforation after sigmoidoscopy of colonoscopy |
| Cost data sources | Centers for Medicare and Medicaid Services: physician Fee Schedule, inpatient prospective payment system and clinical laboratory Fee Schedule |
| year of costing | 2010 |
| Inflation adjustment | Not reported |
| Discount rate | 3% |
| Reported results | In the base case, FIT dominated other strategies. The advantage of FIT over FS and COLO was contingent on rates of uptake and adherence that are well above current US rates. Compared with FIT, FS and COLO both cost < $ 50,000 / QALY gained when FIT per-cycle adherence was < 50 %. COLO cost $ 56,800 / QALY gained vs. FS in the base case. COLO cost < $ 100,000 / QALY gained vs. FS when COLO yielded a relative risk of proximal CRC of < 0.5 vs. no screening. |
| Sensitivity analysis | One-way and probabilistic sensitivity analysis |
| Key variables influencing results | Uptake and adherence, the relative costs of the tests, the costs of CRC care, and the risk of CRC in the population |
| Reported limitations | First, this is a modeling study, not a controlled trial, and the estimates for colonoscopy cannot be validated against data from controlled trials. It is not possible to account for all contingencies in a simulation.  Second, the results for FOBT and FS represent projections validated against data from RCTs, whereas the results for FIT and COLO cannot be validated against comparable studies in which control subjects were not screened.  Third, in reality, people may switch between screening strategies.  Fourth, our model applies to a US population and setting, and may not be generalizable.  Finally, we do not present explicitly scenarios for COLO in which FS as modeled here would clearly be highly preferable; such comparisons are probably not valid, because it is not justifiable to use data from controlled trials of sigmoidoscopy and selected observational studies of colonoscopy to conclude that sigmoidoscopy is superior. |
| Model validation | yes |
| Conclusions | Screening colonoscopy may be cost-effective compared with FIT and sigmoidoscopy, depending on the relative rates of screening uptake and adherence and the protective benefit of colonoscopy in the proximal colon. Colonoscopy’s cost-effectiveness compared with sigmoidoscopy is contingent on the ability to deliver ~ 50 % protection against CRC in the proximal colon. |

Table 26. Data extraction of Ladabaum et al. 2013(29).

| Item | Data |
| --- | --- |
| Author | Ladabaum et al. |
| Year | 2013 |
| Country | United States |
| Perspective | Third party payer (Insurer like Medicare) |
| Time horizon and cycles | Not clearly stated, probably lifetime time horizon |
| Population | 50 – 80 yrs average risk people |
| Intervention(s) | ^m^SEPT9-2well and ^m^SEPT9-3well |
| Comparator (s) | Annual FOBT; Annual FIT; SIGq5Y; COLq10Y; Combination sigmoidoscopy/FOBT; Combination sigmoidoscopy/FIT |
| Modelling approach | Decision analysis model |
| Effectiveness data sources | Published literature including cost-effectiveness analyses, systematics reviews and individual studies |
| Outcomes measures | CRC cases, CRC stage, deaths attributable to CRC, QALYs/person, increment cost/QALY gained |
| Costs | ^m^SEPT9, FOBT, FIT, sigmoidoscopy, sigmoidoscopy with biopsy, colonoscopy, colonoscopy with lesion removal, major hemorrhage after sigmoidoscopy or colonoscopy and perforation sigmoidoscopy or colonoscopy |
| Cost data sources | Centers for Medicare & Medicaid Services: physician Fee Schedule, Inpatient Prospective Payment System, Clinical Laboratory Fee Schedule and estimated |
| year of costing | 2010 |
| Inflation adjustment | Not reported |
| Discount rate | 3% |
| Reported results | Assuming perfect uptake, adherence, and follow-up after abnormal screening, the greatest reductions in colorectal cancer incidence and mortality compared with no screening were observed with sigmoidoscopy/FIT, and the reductions with mSEPT9-3well and mSEPT9-2well were lower than with the alternatives.  Compared with no screening, mSEPT9-2well and mSEPT9-3well yielded costs/QALY gained of $11,500 and $8,400, respectively. In comparison, sigmoidoscopy/FIT, sigmoidoscopy/FOBT, and colonoscopy yielded costs/QALY gained under $3,000, and FOBT, sigmoidoscopy, and FIT were more effective and less costly compared with no screening.  While all strategies yielded substantial gains in life expectancy compared with no screening, and the differences in effectiveness between strategies were comparatively small, annual FIT was preferred in incremental comparisons between strategies. It was more effective and less costly than mSEPT9-2well, mSEPT9-3well, FOBT, sigmoidoscopy, and colonoscopy. |
| Sensitivity analysis | One-way and probabilistic analysis |
| Key variables influencing results | Test performance, screening tests costs, uptake and adherence |
| Reported limitations | The degree of independence between individual tests in a given strategy is not known, and our model assumes independence. We did not model adenoma multiplicity or location, and the number of people with adenomas and colorectal cancer detected by size and stage were not validated in the same way that colorectal cancer incidence and mortality reduction were validated. Disutility’s associated with screening are not included. |
| Model validation | Yes, the made different validated were exercises specified at supplementary material |
| Conclusions | mSEPT9 seems to be effective and cost-effective compared with no screening. To be cost-effective compared with established strategies, mSEPT9 or blood-based biomarkers with similar test performance characteristics would need to achieve substantially higher uptake and adherence rates than the alternatives. It remains to be proven whether colorectal cancer screening with a blood test can improve screening uptake or long-term adherence compared with established strategies.  Our study offers insights into the potential role of colorectal cancer screening with blood-based biomarkers. |
| Comentarios del cliente | This shows how the net impact of an emerging screening test at the population level is likely to depend on the degree to which it induces non screened persons to undergo screening, and the degree to which it changes the fractions of the population taking up each specific strategy.  In a health care system that accepts multiple colorectal cancer screening strategies, the potential effect of screening based on mSEPT9 or comparable biomarkers depends on the extent to which it increases overall screening uptake as opposed to being substituted for current alternatives, and the relative rates of adherence and follow-up compared with current alternatives. For example, if mSEPT9-based screening were taken up only by persons who are currently not screened at all, then net clinical benefits would be realized at acceptable costs. Alternatively, if a test such as mSEPT9-3well with current test performance characteristics were simply substituted for current alternatives without improvements in adherence or follow-up, then overall effectiveness would decrease, and costs would increase. The real effect of screening with mSEPT9 or comparable biomarkers is likely to lie between these extremes. |

Table 27. Data extraction of Hassan et al.2012(30).

| Item | Data |
| --- | --- |
| Author | Hassan et al. |
| Year | 2012 |
| Country | United States |
| Perspective | Third party payer |
| Time horizon and cycles | Lifetime time horizon |
| Population | 50 years |
| Intervention(s) | COL or SIG q10Y or combination of daily aspirin 75 mg with COL or SIG q10Y |
| Comparator (s) | No screening COL or SIG q10Y or combination of daily aspirin 75 mg with COL or SIG q10Y |
| Modelling approach | Markov model |
| Effectiveness data sources | Case-control study, multicenter randomized controlled trial, follow-up of five randomized trials |
| Outcomes measures | CRC cases, CRC prevented, CRC deaths, life-years gained, gain in life expectancy per person, screening cost, care for CRC, total cost per person and ICER |
| Costs | Colonoscopy, colonoscopy with polypectomy, sigmoidoscopy, 75mg Aspirin daily per 1 year, aspirin-related upper gastrointestinal bleeding, aspirin-related hemorrhagic stroke, indirect costs OC, localized CRC treatment, regional CRC treatment, distant CRC treatment, bleeding and perforation |
| Cost data sources | CMS.gov. Centers for Medicare & Medicaid Services |
| year of costing | 2010 |
| Inflation adjustment | 2010 USD using Medical Consumer Price Index |
| Discount rate | 3% |
| Reported results | When comparing endoscopic strategies with the no-screening scenario, sigmoidoscopy and colonoscopy screening appeared to be a cost-effective alternative with an ICER of US$7434 and US $6307 per life-year saved, respectively.  Overall, the combination of aspirin and sigmoidoscopy screening was less costly and more effective than sigmoidoscopy screening only, so that the combined strategy was more cost-effective than sigmoidoscopy (ie, dominated), resulting in a net discounted saving of US$278 per person. The combined strategy was also cost-effective as compared to no screening, with an ICER of US$6511 per life-year saved, as well as when compared with an aspirin (without sigmoidoscopy) strategy.  Colonoscopy strategy (without aspirin) was more effective and cost-effective than the combined sigmoidoscopy and aspirin strategy (ICER: US$33 126). Moreover, the combination of aspirin and colonoscopy screening was costlier and more effective than colonoscopy screening only and was more cost-effective as well (ICER: US$5413). The combined strategy was also cost-effective as compared to no screening, with an ICER of US$6237 per life-year saved, and to aspirin/sigmoidoscopy (ICER: US$21 765, table 2).  Primary prevention with low-dose aspirin appeared to be cost saving as compared to no screening, with the saving in CRC treatment being larger than the expenditure in the drug and drug-related complications. The overall saving was US$436 per person.  When simulating the addition of either sigmoidoscopy or colonoscopy screening to patients already undertaking a primary cardiovascular prevention with low-dose aspirin, such addition appeared to be cost-effective, with an ICER of US$12 509 and US$15 526 per life-year saved, respectively. |
| Sensitivity analysis | One- and two-way sensitivity analysis and probabilistic sensitivity analysis |
| Key variables influencing results | Aspirin related-reduction polyp prevalence  Aspirin-related prevention of proximal CRC  Aspirin-related UGB risk and stroke  Colonoscopy-related CRC prevention rates  Aspirin costs |
| Reported limitations | Estimates on aspirin efficacy were derived from randomized studies on cardiovascular prevention, CRC incidence and death rates not representing primary end points.  Second, suboptimal colonoscopy efficacy in preventing right-sided CRC was extrapolated from a population study, with no randomized studies being available.  Third, we did not assume any aspirin-related reduction of polyp prevalence, potentially underestimating the cost-effectiveness of aspirin addition.  Fourth, we did not include any potential primary prevention of cardiovascular disease, which was outside the purposes of this analysis.  Fifth, we did not compare the endoscopic strategies with or without aspirin with non-endoscopic screening alternatives with the cost effectiveness of the addition of aspirin over endoscopy being independent from the relative cost effectiveness between endoscopic and non-endoscopic strategies.  Finally, we did not assume a suboptimal adherence to endoscopic screening in the reference case scenario, since the cost-effectiveness of the addition of aspirin over endoscopy is independent from such adherence. |
| Model validation | Yes, in order to validate the model against previous data, they compared the model outputs with those of other models |
| Conclusions | In settings where the efficacy of colonoscopy is suboptimal for preventing proximal colon cancer, the addition of low-dose aspirin may be effective and cost-effective. Addition of aspirin loses cost-effectiveness when the efficacy of colonoscopy in preventing proximal colon cancer reaches 73%. |

Table 28. Data extraction of Barouni et al. 2012(31).

| Item | Data |
| --- | --- |
| Author | Barouni et al. |
| Year | 2012 |
| Country | Iran |
| Perspective | Third party payer (provincial ministry of health) |
| Time horizon and cycles | Lifetime;1-year |
| Population | 50-75 years |
| Intervention(s) | gFOBT+SIG q5Y; SIG q5Y; CTC q5Y; Double contrast enema q5Y; Fecal DNA q3Y; Annual iFOBT; COL q10Y; Annual low-S gFOBT ; Annual high-S gFOBT; Low-S gFOBT q2Y |
| Comparator (s) | No screening |
| Modelling approach | Markov model |
| Effectiveness data sources | Published literature |
| Outcomes measures | QALY, costs of screening strategy, CRC prevented, decrease mortality, deaths prevented |
| Costs | Tests costs, cost of cancer care |
| Cost data sources | Published literature |
| year of costing | Not reported |
| Inflation adjustment | Adjusted to USD 2011 |
| Discount rate | 5% |
| Reported results | The incremental cost per quality-adjusted life-year gained for colorectal cancer screening ranged from $654 with annual fecal immunochemical testing through $8700 for colonoscopy every 10 years to $9067 for annual low-sensitivity guaiac fecal occult blood testing. |
| Sensitivity analysis | The one-way and probabilistic sensitivity analyses |
| Key variables influencing results | variations in sensitivity of the test to detect complex adenoma, cost of the test, compliance with screening and cost of colon cancer care. |
| Reported limitations | Model-based economic evaluation depends on the data accessible in the medical text, which is continuously evolving. The natural history of colon cancer is based on assumptions concerning the development from adenoma to carcinoma and the transition time from a low-risk polyp to a malignant neoplasm. We did not include the possibility of regression of polyps. We also did not model malignancies arising from lesions other than polyps.  Other boundaries were related to incorporating the following untested assumptions: characteristics of test performance would remain constant on repeat testing, incidence of adenoma would be impassive by screening and compliance with testing was random.  The model did not incorporate the costs of establishing the infrastructure to implement population-based screening for colorectal cancer. |
| Model validation | Not reported |
| Conclusions | In conclusion, screening of individuals (average risk) for colon cancer is a cost-effective measure, even with less-than-perfect compliance. Recognizing that decisions about screening for colorectal cancer depend on local resources and individual patient preferences, either an annual high sensitivity fecal test, such as a fecal immunochemical test, or colonoscopy each 10 years offer good value for money in Iran. Finally, Annual high-sensitivity fecal occult blood testing, such as a fecal immunochemical test, or colonoscopy every 10 years offer the best value for the money in Iran. |

Table 29. Data extraction of Whyte et al. 2012(33).

| Item | Data |
| --- | --- |
| Author | Whyte et al. |
| Year | 2012 |
| Country | England |
| Perspective | National Health System |
| Time horizon and cycles | Lifetime; 1-year |
| Population | cohort of 30-year-old individuals in the general population of England with normal colon epithelium through to the development of adenomas |
| Intervention(s) | gFOBT at 60–69 years (biennial)  gFOBT at 60–74 years (biennial)  iFOBT at 60, 65, 70 years  iFOBT at 60–69 years (biennial)  iFOBT at 60–74 years (biennial)  FS age 55  FS age 55, 65  FS age 55, gFOBT 66–74 (biennial)  FS age 55, iFOBT 66–74 (biennial)  FSIG age 55, iFOBT 60, 65,70  FS age 55, iFOBT 60–74 (biennial)  FS age 55, iFOBT 56–74 (biennial) |
| Comparator (s) | No screening |
| Modelling approach | State transition model |
| Effectiveness data sources | Estimated within the model calibration process |
| Outcomes measures | CRC detection rates, HR adenoma detection rates, LR adenoma detection rates, false positive rates, cost, Lys, QALYs and  reduction (%) of: CRC incidence, CRC mortality, CRC treatment costs. Endoscopy requirements, harm, number needed to screen. |
| Costs | gFOBT screen and iFOBT for: non-compliers, normal result and positive result, FS screen excluding FS exam (non-compliers), FS screen excluding FS exam (referred to COL), FS (without polypectomy), FS (with polypectomy), Proportion of LR adenomas being referred for COL following FS, COL (without polypectomy), COL (with polypectomy), treating bowel perforation (major surgery), admittance for bleeding (overnight stay on medical ward), Pathology cost for adenoma, Pathology cost for cancer, Lifetime cost – screen-detected: Dukes A, Dukes B, Dukes C and Dukes D. |
| Cost data sources | Southern Hub screening costings model, NHS reference costs, screening center estimates, FS trial data, NHS reference costs, published literature |
| year of costing | Not reported |
| Inflation adjustment | Not reported |
| Discount rate | 3.5% |
| Reported results | All screening strategies evaluated had an incremental cost effectiveness ratio of < £20 000 compared with a strategy of no screening. FS screening at age 55 was associated with the greatest QALY gain; QALY gains are very similar between ages 52 and 58 The greatest reduction in CRC incidence and mortality is seen when a one-off FS screen is offered at age 64. |
| Sensitivity analysis | One-way sensitivity analysis |
| Key variables influencing results | Uptake, endoscopy costs and iFOBT threshold. |
| Reported limitations | As with any health economics model, our analysis incorporates both structural and parametric assumptions which influence the predictions of cost and effectiveness of the screening strategies. The original options appraisal by Tappenden et al. identified the calibrated natural history model parameters as an important area of uncertainty.  The endoscopy costs used in this model are associated with considerable uncertainty, and the sensitivity analyses demonstrated that the expected costs are very sensitive to these values  The model used the common assumption that FOBT characteristics do not vary by screening round (although specificity was allowed to vary by age) but the gFOBT second round data were inconsistent with this assumption.  If significantly higher false positive rates are in fact seen at a second ⁄ repeat screen, then the results presented here may overestimate the benefit of screening strategies involving repeated FOBT screens.  Data on adenoma prevalence by both age and location (proximal ⁄ distal) were not available.  Very limited data were available on transition rates post-polypectomy; hence there is considerable uncertainty surrounding the modelling of surveillance.  This analysis combined data from several countries. |
| Model validation | Yes (calibration) |
| Conclusions | The analysis of a range of screening strategies, compared with the current CRC screening policy in England (biennial gFOBT 60–74), suggests that a change to this policy would produce additional clinical benefits and be cost effective. one screening modality should be considered as these have the potential to provide additional benefits whilst greater patient choice may also increase uptake. The additional endoscopy resources required by such new strategies may influence adoption by the screening programme. |

Table 30. Data extraction of Chauvin et al. 2012(34).

| Item | Data |
| --- | --- |
| Author | Chauvin et al. |
| Year | 2012 |
| Country | France |
| Perspective | Third-party payer |
| Time horizon and cycles | 30 years; 1-year |
| Population | Average risk subjects from 50 years old |
| Intervention(s) | Immunological FOBT, CTC every 10 years, CTC every 5 years |
| Comparator (s) | gFOBT |
| Modelling approach | Markov model |
| Effectiveness data sources | Screening pilot program, meta-analysis, systematic review and published literature |
| Outcomes measures | Positivity rate, number of colonoscopy procedures (due to the screening procedures, due to symptomatic CRC, due to the follow-up of treated patients), number of diagnosed CRC, distribution of CRC according to their stage (stage I, II, III and IV), CRC prevention rate, total cost, life-years gained, ICER |
| Costs | Guaiac test, immunological test, virtual colonoscopy, optical colonoscopy, optical colonoscopy with polypectomy of non-adenomatous polyps, optical colonoscopy with polypectomy of adenomas (50–69/C70 years old), optical colonoscopy with diagnosis of cancer (50–69/C70 years old), complication (bleeding or perforation), treatment costs of colorectal cancer stage I, treatment costs of colorectal cancer stage II, treatment costs of colorectal cancer stage III, treatment costs of colorectal cancer stage IV |
| Cost data sources | Published literature including individua articles and cost-effectiveness studies, Official French coding |
| year of costing | 2007 |
| Inflation adjustment | the medical care component of the consumer price index available at INSEE Web site (103.42 for 2006 costs, 103.8 for 2007 costs, 103.4 for 2004 costs) |
| Discount rate | 3% |
| Reported results | Biennial guaiac FOBT yielded the lowest positivity rate with 2.85% of positive tests, while, at the opposite, CTC10.  The reference strategy, guaiac FOBT, was the least expensive and the least effective.  had the greatest rate with 12.01%. The range of WTP values for which each strategy can be optimal is described in Table 4. gFOBT was optimal until the WTP of the third-party payer reached 760 €/LYG. This WTP corresponds to the ICER of moving from the reference strategy to CTC10. Below this threshold, no alternative strategy generated health gains that could offset its extra cost. From a WTP of 760 €/LYG to 8,063 €/LYG, CTC10 became the optimal strategy. Finally, CTC5 saved more discounted life-years than CTC10 and yielded the highest INB from a WTP of 8,063 €/LYG. |
| Sensitivity analysis | One-way and probabilistic sensitivity analysis |
| Key variables influencing results | Discount rate, time horizon, participation rates |
| Reported limitations | We did not include sigmoidoscopy.  One limitation concerns data, which are not obtained from a systematic literature review but rather rest on selected and prominent research published in medical journals.  Another strong assumption relates to the adherence rate to screening. The base-case model used French data from FOBT screening campaigns.  A series of limitations specifically relate to CTC strategies. A first one has to do with the CTC screening techniques which include radiological investigations and thus expose patients to radiations. Radiological side effects have not been taken into account, though admittedly they affect both the cost and the effectiveness dimensions of the analysis. A second limitation associated with CTC screening relates to extracolonic findings during the clinical investigation. They are not considered here. |
| Model validation | Yes, Validation of the model was carried out by comparing simulation results with actual data obtained from an INVS (the French institute for public health surveillance) study and from published clinical trials’ data. |
| Conclusions | When there is a high level of resources and a corresponding willingness to dedicate it to a CRC screening program, biennial immunological FOBT and CTC5 strategies are significantly more effective and more expensive than the biennial guaiac test. These two strategies provided very close expected net benefits, which prevents the costeffectiveness analysis from setting them apart. However, differences remained as these strategies involved very different organizational patterns. |

Table 31. Data extraction of Wang et al. 2012(35).

| Item | Data |
| --- | --- |
| Author | Wang et al. |
| Year | 2012 |
| Country | China |
| Perspective | Not stated |
| Time horizon and cycles | 1-year |
| Population | Chinese individuals aged from 50 to 80 year |
| Intervention(s) | Single COLO and repeat COLO |
| Comparator (s) | No screening |
| Modelling approach | Markov model |
| Effectiveness data sources | Published literature |
| Outcomes measures | Total number of non-advanced CRA cases, total number of advanced CRA cases, total number of early CRC cases, total number of advanced CRC cases, Cases of CRC prevented, proportion of CRC case prevented (%), total number of early CRC-related dead cases, total number of advanced CRC-related dead cases, total loss of CRC-related life years, effect (life year), life-years saved, number of procedures (CSPY, bleeding, perforation, therapeutic with polypectomy), costs (CSPY (including complications), polypectomy (including complications), early CRC, advanced CRC, total costs), C/E, ICER. |
| Costs | Colonoscopy, polypectomy, bleeding, perforation, treatment for the early CRC, CT scan, colorectal radical resection, hospital charges (9 days), treatment for the late CRC, CT scan, colorectal enlarged radical resection, PET scan, metastatic disease on liver, hospital charges 9 days (up to 30 days), chemotherapy: FOLFOX for 6 months_ Shanghai. |
| Cost data sources | Shanghai medical health care services and prices assembly (2010) Shanghai municipal health bureau. |
| year of costing | 2010 |
| Inflation adjustment | Not reported |
| Discount rate | 3% |
| Reported results | Assuming a first-time compliance rate of 90%, repeat screening colonoscopy and single colonoscopy can reduce the incidence of colorectal cancer by 65.8% and 67.2% respectively. The incremental cost-effectiveness ratio for single colonoscopy (49 Renminbi Yuan [RMB]) was much lower than that for repeat screening colonoscopy (474 RMB). Single colonoscopy was a more cost-effective strategy, which was not sensitive to the compliance rate of colonoscopy and the cost of advanced colorectal cancer. |
| Sensitivity analysis | One-way sensitivity analysis |
| Key variables influencing results | Compliance rates and treatment cost for advanced CRC |
| Reported limitations | The primary shortcoming is no sensitivity analysis for age in our study due to lack of age distribution in Chinese population. Because the incidence rate of CRC shows an age-dependent increase, the number of cancers prevented per single CSPY is higher in the older than in the younger. Screening by a single colonoscopy is far more likely to lose its preventive power if scheduled too early.  Secondly, although our clinical data were based on data mainly from China, some data from Europe and the USA had been used because of the data unavailable in China. Thirdly, indirect costs were not included.  Finally, the single CSPY based strategy was suggested to be the more cost-effective strategy for screening and management of colorectal neoplasm and may be recommended in China clinical practice. |
| Model validation | Not reported |
| Conclusions | Single colonoscopy is suggested to be the more cost-effective strategy for screening and management of colorectal neoplasms and may be recommended in China clinical practice. |

Table 32. Data extraction of Pinzon Florez et al. 2012(36).

| Item | Data |
| --- | --- |
| Author | Pinzon Florez et al. |
| Year | 2012 |
| Country | Colombia |
| Perspective | Third-party payer |
| Time horizon and cycles | 76 years old or death;1-year |
| Population | 20 years old |
| Intervention(s) | gFOBT biennial, gFOBT annual, Immunological FOBT biennial, Immunological FOBT annual, sigmoidoscopy 5 years + Immunological FOBT annual, COLO 10 years, sigmoidoscopy 5 years |
| Comparator (s) | No screening |
| Modelling approach | Markov model |
| Effectiveness data sources | Not reported |
| Outcomes measures | Costs, incremental costs, QALYs, incremental effectiveness, C/E, ICER |
| Costs | direct costs associated with interventions in evaluation, management of adverse events associated with these, treatment of polyposis and cancer in its different stages |
| Cost data sources | Resources costs: Manual tarifario oficial del seguro obligatorio de accidentes de tránsito (SOAT) de Colombia and for the treatment costs la base de datos del sistema de información de precios de medicamentos (SISMED) del Ministerio de Salud de Colombia |
| year of costing | Not reported |
| Inflation adjustment | USD 2010 using official exchange rates |
| Discount rate | 3% |
| Reported results | The screening strategy more cost-effective was the gFOBT biennial type. The cost per life year gained was US$10,347.37, US$18,380.64, and US$45,158.05. For gFOBT biennial, gFOBT annual and FOBT immunological biennial respectively. |
| Sensitivity analysis | One-way and probabilistic sensitivity analysis |
| Key variables influencing results | Cost of strategies, percentage of false positives and follow-up |
| Reported limitations | Not stated |
| Model validation | Not reported |
| Conclusions | In conclusion, from the perspective of the third payer, our study showed that the strategy FOBT biennial matter is efficient for the Colombian context. The efficiency of the test depends on the disease burden and specificity of the test. The combination FOBT and sigmoidoscopy can be efficient, as long as the price of the test is guaranteed, as well as the coverage and follow-up of positive individuals. |

Table 33. Data extraction of Sharp et al. 2012(37).

| Item | Data |
| --- | --- |
| Author | Sharp et al. |
| Year | 2012 |
| Country | Ireland |
| Perspective | Health care payer perspective (Health Service Executive (HSE)) |
| Time horizon and cycles | 100 years old or death; 1-year |
| Population | 30 years old |
| Intervention(s) | gFOBT at 55 – 74 years  gFOBT at 55 – 64 years  gFOBT at 65 – 74 years  FIT at 55 –74 years  FIT at 55 –64 years  FIT at 65 –74 years  FSIG once at 60 years  FSIG once at 55 years |
| Comparator (s) | No screening |
| Modelling approach | Markov model |
| Effectiveness data sources | Published literature |
| Outcomes measures | Cost of screening and CRC per person, incremental costs, expected QALYs per person, incremental QALYs per person, ICER-Incremental cost per QALY gained |
| Costs | gFOBT kit, gFOBT processing/analysis, FIT kit, FIT processing/analysis, cost of FSIG (with/without polypectomy), cost of COL, cost of CTC, cost of treating bowel perforation, cost of admittance for bleeding, pathology cost for adenoma, pathology cost for cancer, lifetime cost stage I, II, III and IV CRC-symptomatic and lifetime cost stage I, II, III and IV CRC-screen-detected. |
| Cost data sources | Estimated by authors, VHI Healthcare, HSE Casemix Unit, 2008, expert opinion and published literature |
| year of costing | 2008 |
| Inflation adjustment | Costs were converted to 2008 Euros and inflated using the consumer price index for health |
| Discount rate | 4% |
| Reported results | All scenarios would be considered highly cost-effective compared with no screening. The lowest incremental cost-effectiveness ratio was found for FSIG, followed by FIT (€1696) and gFOBT (€4428); gFOBT was dominated.  Compared with FSIG, FIT was associated with greater gains in QALYs and reductions in lifetime cancer incidence and mortality, but was costlier, required considerably more colonoscopies and resulted in more complications. |
| Sensitivity analysis | One-way and probabilistic sensitivity analysis |
| Key variables influencing results | Discount rate, costs of screening tests and costs of managing colorectal cancer |
| Reported limitations | Unlike most previous natural history models, we assumed that some cancers (14%) would arise without a prior adenoma. Our model is likely to have underestimated screening effectiveness, with the extent of underestimation differing for faecal and endoscopic tests.  Thus, our study (and other similar studies) may somewhat overestimate benefits of screening.  In common with similar analyses, we did not include costs of setting up programme infrastructure and some costs associated with ongoing programme administration and delivery. Many of these depend on the business model adopted. Because of the limited evidence base, costs incurred by screening participants (such as travel costs) and societal costs (such as lost productivity) were not included.  A major area of uncertainty in this and other similar models relates to the true underlying population prevalence of adenomas.  Finally, although cost-effectiveness analysis is a valuable tool for comparing costs and benefits of alternative screening options, it may not fully address ‘real world’ issues around programme implementation and delivery. |
| Model validation | Not reported |
| Conclusions | This analysis suggests that a screening programme based on biennial screening at 55–74 years with FIT would be preferable to one based on biennial gFOBT (with reflex FIT) at 55–74 years or once-only FSIG at 60 years. Although a programme based on FIT is expected to result in the greatest health improvement, it would require more colonoscopy resources and result in more individuals suffering adverse effects. The major challenges for policy makers are, therefore, balancing the benefits and harms of screening while ensuring sufficient capacity for follow-up of screen-detected adenomas and cancers. |

# References

1. Lew J Bin, St. John DJB, Macrae FA, Emery JD, Ee HC, Jenkins MA, et al. Evaluation of the benefits, harms and cost-effectiveness of potential alternatives to iFOBT testing for colorectal cancer screening in Australia. Int J Cancer. 2018;143(2):269–82.

2. Van der Meulen MP, Lansdorp-Vogelaar I, Goede SL, Kuipers EJ. Cost- effectiveness of Colonoscopy versus CT Colonography Screening with participation rates and costs. 2018;000(0):1–11.

3. Melnitchouk N, Soeteman D, Davids JS, Fields A. Cost-effectiveness of colorectal cancer screening in Ukraine. Epidemiol Rev. BioMed Central; 2011;33(1):88–100.

4. Greuter MJE, De Klerk CM, Meijer GA, Dekker E, Coupe VMH. Screening for colorectal cancer with fecal immunochemical testing with and without postpolypectomy surveillance colonoscopy: A cost-effectiveness analysis. Ann Intern Med. 2017;167(8):544–54.

5. Aronsson M, Carlsson P, Levin L, Hager J, Hultcrantz R. Cost-effectiveness of high-sensitivity faecal immunochemical test and colonoscopy screening for colorectal cancer. Br J Surg. 2017;104(8):1078–86.

6. Goede SL, Rabeneck L, Van Ballegooijen M, Zauber AG, Paszat LF, Hoch JS, et al. Harms, benefits and costs of fecal immunochemical testing versus guaiac fecal occult blood testing for colorectal cancer screening. PLoS One. 2017;12(3):1–15.

7. Coldman A, Flanagan W, Nadeau C, Wolfson M, Fitzgerald N, Memon S, et al. Projected effect of fecal immunochemical test threshold for colorectal cancer screening on outcomes and costs for Canada using the OncoSim microsimulation model. J Cancer Policy. Elsevier; 2017;13(July):38–46.

8. Atkin W, Brenner A, Martin J, Wooldrage K, Shah U, Lucas F, et al. The clinical effectiveness of different surveillance strategies to prevent colorectal cancer in people with intermediate-grade colorectal adenomas: A retrospective cohort analysis, and psychological and economic evaluations. Health Technol Assess (Rockv). 2017;21(25).

9. Murphy J, Halloran S, Gray A. Cost-effectiveness of the faecal immunochemical test at a range of positivity thresholds compared with the guaiac faecal occult blood test in the NHS Bowel Cancer Screening Programme in England. BMJ Open. 2017;7(10):1–10.

10. M.J.E. G, J. B, R.J.A. F, E. D, J.-B. L, G.A. M, et al. The potential of imaging techniques as a screening tool for colorectal cancer: A cost-effectiveness analysis. Br J Radiol. 2016;89(1063):20150910.

11. Ladabaum U, Mannalithara A. Comparative Effectiveness and Cost Effectiveness of a Multitarget Stool DNA Test to Screen for Colorectal Neoplasia. Gastroenterology. Elsevier, Inc; 2016;151(3):427–439.e6.

12. Wong MCS, Ching JYL, Chan VCW, Lam TYT, Luk AKC, Wong SH, et al. Colorectal cancer screening based on age and gender: A cost-effectiveness analysis. Med (United States). 2016;95(10):1–7.

13. Espinola N, Maceira D, Palacios A. Costo-efectividad de las pruebas de tamizaje del cáncer colorrectal en la Argentina. 2016;46(1):8–17.

14. Hassan C, Rex DK, Zullo A, Kaminski MF. Efficacy and cost-Effectiveness of screening colonoscopy according to the adenoma detection rate. United Eur Gastroenterol J. 2015;3(2):200–7.

15. Wong MCS, Ching JY, Chan VC, Sung JJ. The comparative cost-effectiveness of colorectal cancer screening using faecal immunochemical test vs. colonoscopy. Sci Rep. Nature Publishing Group; 2015;5(April):1–9.

16. Wong CKH, Lam CLK, Wan YF, Fong DYT. Cost-effectiveness simulation and analysis of colorectal cancer screening in Hong Kong Chinese population: Comparison amongst colonoscopy, guaiac and immunologic fecal occult blood testing. BMC Cancer. BMC Cancer; 2015;15(1):1–12.

17. Sekiguchi M, Igarashi A, Matsuda T, Matsumoto M, Sakamoto T, Nakajima T, et al. Optimal use of colonoscopy and fecal immunochemical test for population-based colorectal cancer screening: A cost-effectiveness analysis using Japanese data. Jpn J Clin Oncol. 2016;46(2):116–25.

18. Hassan C, Gralnek IM. Cost-effectiveness of “full spectrum endoscopy” colonoscopy for colorectal cancer screening. Dig Liver Dis. Editrice Gastroenterologica Italiana; 2015;47(5):390–4.

19. Huang W, Liu G, Zhang X, Fu W, Zheng S, Wu Q, et al. Cost-effectiveness of colorectal cancer screening protocols in urban Chinese populations. PLoS One. 2014;9(10).

20. Ladabaum U, Alvarez-Osorio L, Rösch T, Brueggenjuergen B. Cost-effectiveness of colorectal cancer screening in Germany: current endoscopic and fecal testing strategies versus plasma methylated Septin 9 DNA. Endosc Int Open. 2014;02(02):E96–104.

21. Lejeune C, Le Gleut K, Cottet V, Galimard C, Durand G, Dancourt V, et al. The cost-effectiveness of immunochemical tests for colorectal cancer screening. Dig Liver Dis. Editrice Gastroenterologica Italiana; 2014;46(1):76–81.

22. Hashimoto Y, Igarashi A, Miyake M, Iinuma G, Fukuda T, Tsutani K. Cost-effectiveness analysis of CT colonography for colorectal cancer screening program to working age in Japan. Value Heal Reg Issues. Elsevier; 2014;3(1):182–9.

23. Dinh T, Ladabaum U, Alperin P, Caldwell C, Smith R, Levin TR. Health Benefits and Cost-effectiveness of a Hybrid Screening Strategy for Colorectal Cancer. Clin Gastroenterol Hepatol. Elsevier Inc.; 2013;11(9):1158–66.

24. M. B, H. G, Barouni M, Ghaderi H, Shahmoradi MK. The economic evaluation of screening for colorectal cancer: Case of Iran. Clin Lab. 2013;59(5–6):667–74.

25. Pence BC, Belasco EJ, Lyford CP. Combination aspirin and/or calcium chemoprevention with colonoscopy in colorectal cancer prevention: cost-effectiveness analyses. Surveill Soc. 2017;15(3–4):596–608.

26. Sharp L, Tilson L, Whyte S, Ceilleachair AO, Walsh C, Usher C, et al. Using resource modelling to inform decision making and service planning: the case of colorectal cancer screening in Ireland. BMC Health Serv Res. 2013 Dec 19;13(1):105.

27. Goede SL, Van Roon AHC, Reijerink JCIY, Van Vuuren AJ, Lansdorp-Vogelaar I, Habbema JDF, et al. Cost-effectiveness of one versus two sample faecal immunochemical testing for colorectal cancer screening. Gut. 2013;62(5):727–34.

28. Sharaf RN, Ladabaum U. Comparative effectiveness and cost-effectiveness of screening colonoscopy vs. Sigmoidoscopy and alternative strategies. Am J Gastroenterol. Nature Publishing Group; 2013;108(1):120–32.

29. Ladabaum U, Allen J, Wandell M, Ramsey S. Colorectal cancer screening with blood-based biomarkers: Cost-effectiveness of methylated septin 9 DNA versus current strategies. Cancer Epidemiol Biomarkers Prev. 2013;22(9):1567–76.

30. Hassan C, Rex DK, Cooper GS, Zullo A, Launois R, Benamouzig R. Primary prevention of colorectal cancer with low-dose aspirin in combination with endoscopy: a cost-effectiveness analysis. Gut. C. Hassan, Ospedale Nuovo Regina Margherita, Gastroenterologia ed Endoscopia Digestiva, Via Morosini 30, 00153, Roma, Italy. E-mail: cesareh@hotmail.com: BMJ Publishing Group (Tavistock Square, London WC1H 9JR, United Kingdom); 2012 Aug;61(8):1172–9.

31. Barouni M, Larizadeh MH, Sabermahani A, Ghaderi H. Markov’s Modeling for Screening Strategies for Colorectal Cancer. Asian Pacific J Cancer Prev. M.H. Larizadeh, Research Center for Health Services Management, Kerman University of medical Sciences, Iran, Islamic Republic of. E-mail: mohsenbarouni@yahoo.com: Asian Pacific Organization for Cancer Prevention (268/1 Rama 6 Rd, Rajchathewee, Bangkok 10400, Thailand); 2012 Oct 31;13(10):5125–9.

32. Lucidarme O, Cadi M, Berger G, Taieb J, Poynard T, Grenier P, et al. Cost-effectiveness modeling of colorectal cancer: Computed tomography colonography vs colonoscopy or fecal occult blood tests. Eur J Radiol. Elsevier Ireland Ltd; 2012;81(7):1413–9.

33. Whyte S, Chilcott J, Halloran S. Reappraisal of the options for colorectal cancer screening in England. Color Dis. 2012;14(9).

34. Chauvin P, Josselin JM, Heresbach D. Incremental net benefit and acceptability of alternative health policies: A case study of mass screening for colorectal cancer. Eur J Heal Econ. 2012;13(3):237–50.

35. Wang Z-H, Gao Q-Y, Fang J-Y. Repeat Colonoscopy Every 10 Years or Single Colonoscopy for Colorectal Neoplasm Screening in Average-risk Chinese: A Cost-effectiveness Analysis. Asian Pacific J Cancer Prev. 2012;13(5):1761–6.

36. Pinzon Florez CE, Rosselli D, Gamboa Garay OA. Análisis de Costo-Efectividad de las Estrategias de Tamización de Cáncer Colorrectal en Colombia. Value Heal Reg Issues. 2012;1(2):190–200.

37. Sharp L, Tilson L, Whyte S, O’Ceilleachair A, Walsh C, Usher C, et al. Cost-effectiveness of population-based screening for colorectal cancer: A comparison of guaiac-based faecal occult blood testing, faecal immunochemical testing and flexible sigmoidoscopy. Br J Cancer. 2012;106(5):805–16.
