# Supplementary material for: Economic evaluations of screening strategies for the early detection of colorectal cancer in the average-risk population: A systematic literature review
Source: PLoS One. 2019 Dec 31;14(12):e0227251. doi: 10.1371/journal.pone.0227251 (PMC6938313; doi:10.1371/journal.pone.0227251)
Supplement: S1 Table — (DOCX) [file pone.0227251.s005.docx]

**S3 Table. Data extraction: main characteristics of included studies**

| **Author** | **Country** | **Target population** | **Interventions and comparators** | **Study perspective** | **Model type** | **Main outcomes** | **Sensitivity analyses** |
| --- | --- | --- | --- | --- | --- | --- | --- |
| Lew et al. 2018 | Australia | General population 20y | FIT; pDNA; fDNA; COL; FS; CTC; FS+ FIT; COL +FIT; FIT+FS; FIT+pDNA; no screening. | Health care payer | Microsimulation model | Compared to no screening, all strategies were cost-effective. Only 2 yearly FIT was t cost-effective in all three adherence scenarios. | One-way sensitivity analysis. Scenario analysis.  Supplementary analysis. |
| Van der Meulen et al. 2018 | Netherlands | General population 50–75 y | CTC; COL; no screening | Third party health-care payer | Microsimulation model | With a 100% participation rate, COL dominated CTC. With observed participation, COL with one or two lifetime screenings was less costly than and as effective as the same CTC strategies. With more lifetime screening, CTC dominated COL. | One-way sensitivity analysis and scenario analysis |
| Melnitchouck et al. 2018 | Ukraine | General popultaion 50y | FOBT; FS+ FOBT; COL; no screening | Health care payer | State-transition Markov model | All three screening strategies were cost saving compared to no screening. COL 10- yearly was the dominant strategy compared to no screening with standard adherence to treatment. | One-way, Two-way and three-way sensitivity analysis. |
| Greuter et al. 2017 | Netherlands | General population 55-75y | FIT; FIT+COL; no screening | Health care payer | Microsimulation model | Adding surveillance to FIT screening is not cost effective and substantially increases colonoscopy demand. | 1-way sensitivity analysis |
| Aronsson et al. 2017 | Sweden | General population 60y | FIT; COL; no screening | Health care payer | State-transition Markov model | All screening strategies were cost-effective compared with no screening. Repeated and single screening strategies with COL were more cost-effective than FIT. | Two- way sensitivity analyses |
| Goede et al. 2017 | Canada | general population 40y at average risk of CRC | FIT; gFOBT; no screening | Third party health-care payer | Microsimulation model | FIT every year between age 45±80 years would be the preferred strategy, providing 49 QALYs per 1,000 participants. | One-way sensitivity analysis |
| Coldman et al. 2017 | Canada | Cohort aged 45 years; screening between 50–74 years | FIT; no screening | Third party health-care payer | Microsimulation model (The OncoSim Model) | Biennial FIT was projected to be cost-effective at all thresholds considered. Demand for COL varied strongly with FIT threshold and was greatest for the lowest threshold (50 ng/ml). | Scenario analysis (threshold variation) |
| Murphy et al. 2017 | England | Individuals 60 y | FIT; gFOBT | Health care payer | State-transition Markov model | FIT dominated gFOBT at all thresholds. Nevertheless, at lower thresholds, FIT was associated with substantial increase in COL requirements | Probabilistic sensitivity analysis and one-way sensitivity analysis |
| Greuter et al. 2016 | Netherlands | Healthy individuals 20y | FIT; CTC; MRC; COL | Health care payer | Microsimulation model | All imaging-based strategies (MRC, CTC) were cost-effective compared with no screening. FIT screening was the dominant screening strategy, leading to most LYG and highest cost-savings. Compared with three rounds of COL screening, CTC with five rounds was found to be cost-effective in an incremental analysis of imaging strategies. | One-way sensitivity analysis |
| Ladabaum et al. 2016 | United States | Individuals 50 y | FIT + MT-sDNA | Third party health-care payer | State-transition Markov model | With optimal adherence, yearly FIT and colonoscopy every 10 years were dominant vs. MT-sDNA every 3 years. | One- way and multiway sensitivity analyses |
| Wong et al. 2016 | China | Individuals 50 y | FS;COL | Not reported | State-transition Markov model | When compared with no screening, strategy 5 had the lowest ICER (US$42,515), followed by strategy 3 (US$43,517), strategy 2 (US$43,739), strategy 4 (US$47,710), and strategy 1 (US$56,510). | 1-way sensitivity analyses and probabilistic analysis |
| Espinola et al. 2016 | Argentina | General population of average risk | FIT+ colonoscopia | Third party health-care payer | State-transition Markov model | The most cost-effective strategy was annual FIT.The incremental cost effectiveness ratio (ICER) of FIT versus no intervention was of 980.5 pesos per QALY. | 1-way sensitivity analysis |
| Hassan et al. 2015 | United States | 50-80 years | Low-ADR + Average-ADR + High-ADR + colonoscopia | Not reported | State-transition Markov model | ICER vs no screening, $ per life-year saved – 4,424 | Two-way sensitivity analysis |
| Wong et al. 2015 | China | Individuals 50 y | g-FOBT+FIT+Colonoscopia | Health care payer | State-transition Markov model | Compared with no screening, the ICER presented $20,542/LYs and $3155/QALYs gained for annual FIT, and $19,838/LYs gained and $2976/QALYs gained for biennial FIT. The optimal screening strategy was annual FIT that attained the highest ICER at the threshold of $50,000 per LYs or QALYs gained. | Deterministic (univariate and multivariate) and probabilistic sensitivity analysis (PrSA) were performed |
| Sekiguchi et al. 2015 | Japan | Average-risk population aged 40y or over | FIT; COL; FIT+COL; no screening | Health care payer | State-transition Markov model | All three strategies dominated no screening. Annual FIT (40y)+ COL (50y) dominated annual FIT. Within the Japanese threshold (JPY 5–6 million per QALY gained), COL 10-yearly was the most cost-effective, followed by annual FIT- COL. Considering cost-effectiveness, safety, and the TCS capacity issue together,the authors postulate that FIT-COL would be an optimal strategy when COL is performed ithin the age range 45–55y. | Scenario analyses and probabilistic sensitivity analysis |
| Hassan et al. 2015 | United States | General population 50-100y | SFV-COL ; FUSE- COL; no screening | Social perspective | State-transition Markov model | All strategies were cost-effective compared to no screening. Standard COL was dominated by FUSE-COL | One- and two- way sensitivity analysis |
| Huang et al. 2014 | China | Average risk asymptomatic individuals 40y | FOBT+HRFQ | Third party health-care payer | State-transition Markov model | FOBT+ HRFQ is preferable as an initial screening instrument. Annual FOBT +HRFQ screening is recommended for those who have a negative initial result and those who have a positive result but have failed to comply with colonoscopy procedures. Repeated colonoscopy should be performed at a ten-year interval. | One-way and two-way sensitivity analyses |
| Ladabaum et al. 2014 | Germany | Average risk individuals 50-75y | COL; FOBT; mSept9; FOBT/COL; FIT/COL; FIT; no screening | Health care payer | State-transition Markov model | All strategies were cost-saving compared to no-screening, except for mSEPT9- 2 well 2-yearly, mSEPT9- 2 well yearly and mSEPT9- 3 well yearly, that were deemed cost-effective. FIT dominated m-SEPT9 strategies. FIT was preferred in 49% and FIT/COLO 55,65 in 47% of iterations. | One-way and probabilistic analysis |
| Leujene et al. 2014 | France | Average risk individuals 50-74y | FIT; g-FOBT | Health care payer | State-transition Markov model | FIT were efficient strategies compared to g-FOBT. When all 15 strategies were compared with each other, only five of them remained efficient: the one and two-stool sample Magstream, the one- and two-stool sample FOB-Gold with the 176 ng/mL cut-off, and the two-stool sample OC-Sensor with the 150 ng/mL cut-off. | deterministic sensitivity analyses |
| Hasshimoto et al. 2014 | Japan | Individuals 40y | FOBT+CTC+COL; FOBT+COL; FOBT+ CTC/COL | Health care payer | State-transition Markov model | Strategy 3 dominated strategy 1, while strategy 2 was deemed cost-effective compared to strategy 1. Adding CTC into the current scrrening program (FOBT followed by COL) would be cost-effective. | Scenario and probabilistic sensitivity analyses |
| Dinh et al. 2013 | United States | Individuals 50-75y | FS; FIT; FIT+COL; FIT+FS; COL; no screening | Third party health-care payer | Archimedes model | All strategies dominated no screening. FS was dominated. Annual or biennial FIT (50-65 y)+ COL at 66 years old, delivers clinical and economic outcomes similar to those of CRC screening by single-modality strategies, with a favorable impact on resources demand. | one-way sensitivity analyses |
| Barouni et al. 2013 | Iran | average risk individuals 50 y | gFOBT; FIT; COL; no screening | Third party health-care payer | State-transition Markov model | All strategies were cost-effective compared to no screening. FIT and COL offered the best value for money yeldying an ICER of 654 US$/QALY and 8,700 US$ /QALY vs. no screening, respectively. | One-way and probabilistic sensitivity analyses |
| Pence et al. 2013 | United States | Individuals 50y | COL; Aspirin + COL; Calcium +COL; Aspirin + Calcium + COL | Not reported | State-transition Markov model | COL alone dominated no screening. All remeining strategies were deemed cost-effective compared to no screening. COL+aspirin (ICER $12,950/LYS) or calcium (ICER $13,041/LYS) were the next most cost-effective strategies. | One- and two-way sensitivity analysis |
| Goede et al. 2013 | Netherlands | Individuals 45-80 y | FIT | Health care payer | Microsimulation model | When all screening intervals and age ranges were considered, intensifying screening with one-sample FIT provided equal or more LYG at lower costs compared with two-sample FIT. | Scenario analysis |
| Sharaf et al. 2013 | United States | average risk individuals 50 y | FOBT; FIT; COL; FS+FIT; FS | Third party health-care payer | State-transition Markov model | All strategies except COL dominated no screening. In the base case, FIT dominated other strategies while COL cost $ 56,800/QALY gained vs. FS. The advantage of FIT over FS and COL was contingent on rates of uptake and adherence. Compared with FIT, FS and COL both cost < $ 50,000 / QALY gained when FIT per-cycle adherence was < 50 %. | One-way and probabilistic sensitivity analysis |
| Ladabaum et al. 2013 | United States | average risk individuals 50-80y | Msept9; FOBT; FIT; FS; COL; FS+FOBT; FS+FIT | Third party health-care payer | Decision analysis model | Compared with no screening, mSEPT9-2well and mSEPT9-3well yielded costs/QALY gained of $11,500 and $8,400, respectively. FS/FIT, FS/FOBT, and COL yielded costs/QALY gained under $3,000, while FOBT, sigmoidoscopy, and FIT dominated no screening. FIT was the preferred strategy. | One-way and probabilistic analysis |
| Hassan et al. 2012 | United States | average risk individuals 50 y | COL; FS; COL+Asp; FS+ Asp; no screening | Third party health-care payer | State-transition Markov model | All strategies were cost-effective compared to no screening. COL+ aspirin was the most cost-effective strategy (US$ 6237/LYG), being more cost-effective than COL alone (US$ 6,307/LYG), FS alone (US$ 7,434/LYG), FS+ aspirin (US$ 6,511/LYG) and no screening. | One- and two-way sensitivity analysis and probabilistic sensitivity analysis |
| Barouni et al. 2012 | Iran | individuals 50 y at average risk for CRC | gFOBT; COL; FS; fDNA; FIT; Double contrast barium enema; FOBT+FS | Third party health-care payer | State-transition Markov model | The incremental cost per quality-adjusted life-year gained for colorectal cancer screening ranged from $ 654 with annual FIT through $ 8,700 for COL-10 yearly, to $ 9,067 for annual low-S gFOBT. | One-way and probabilistic sensitivity analyses |
| Whyte et al. 2012 | England | General population (30 years old) | No screening; g-FOBT; FIT; FS; FS+FIT; FS + gFOBT | Health care payer | State transition model | All screening strategies had an ICER < £20 000 compared to no screening. Biennial screening with gFOBT or FIT were cost saving compared with no screening, with biennal FIT dominating gFOBT. FS at age 55, followed by biennall FIT (56-74) was associated with the greatest net monetary benefit, reduction in CRC incidence, mortality and treatment costs. | One-way sensitivity analysis |
| Chauvin et al. 2012 | France | 50 - 80yrs average risk subjects | g-FOBT; FIT; CTC | Third party health-care payer | State-transition Markov model | The reference strategy, guaiac FOBT, was the least expensive and the least effective. FIT was dominated by CTC5gFOBT was optimal until the WTP of the third-party payer reached 760 €/LYG. This WTP corresponds to the ICER of moving from the reference strategy to CTC10. From a WTP of 760 €/LYG to 8,063 €/LYG, CTC10 became the optimal strategy. Finally, CTC5 saved more discounted life-years than CTC10 and yielded the highest INB from a WTP of 8,063 €/LYG. | One-way and probabilistic sensitivity analysis |
| Wang et al. 2012 | China | average risk 50-80 years individuals | COL; no screening | Not reported | State-transition Markov model | Compared to no screening, the ICER for single COL was more cost-effective than COL 10-yearly (49 Renminbi Yuan [RMB] vs. 474 RMB). | One-way sensitivity analysis |
| Pinzon Florez et al. 2012 | Colombia | average risk individuals aged 20 years | gFOBT; FIT; COL; FS; FS+ gFOBT; FS+ FIT; no screening | Third party health-care payer | State-transition Markov model | The biennal gFOBT was the most cost-effecive. The cost per life year gained was US$10,347.37, US$18,380.64, and US$45,158.05. for biennal gFOBT , annual gFOBT and biennal FIT respectively. | One-way and probabilistic sensitivity analysis |
| Sharp et al. 2012 | Ireland | General population (30 years old) | FIT; gFOBT + FIT; FS; no screening | Health care payer | State-transition Markov model | All strategies were highly cost-effective compared to no screening. The lowest ICER vs no screening (€589/QALY) was found for FS, followed by FIT (1696) and gFOBT (4428); gfFOBT was dominated. | One-way and probabilistic sensitivity analysis |
