# Supplementary figures and images for: Economic evaluations of screening strategies for the early detection of colorectal cancer in the average-risk population: A systematic literature review
Source: PLoS One. 2019 Dec 31;14(12):e0227251. doi: 10.1371/journal.pone.0227251 (PMC6938313; doi:10.1371/journal.pone.0227251)

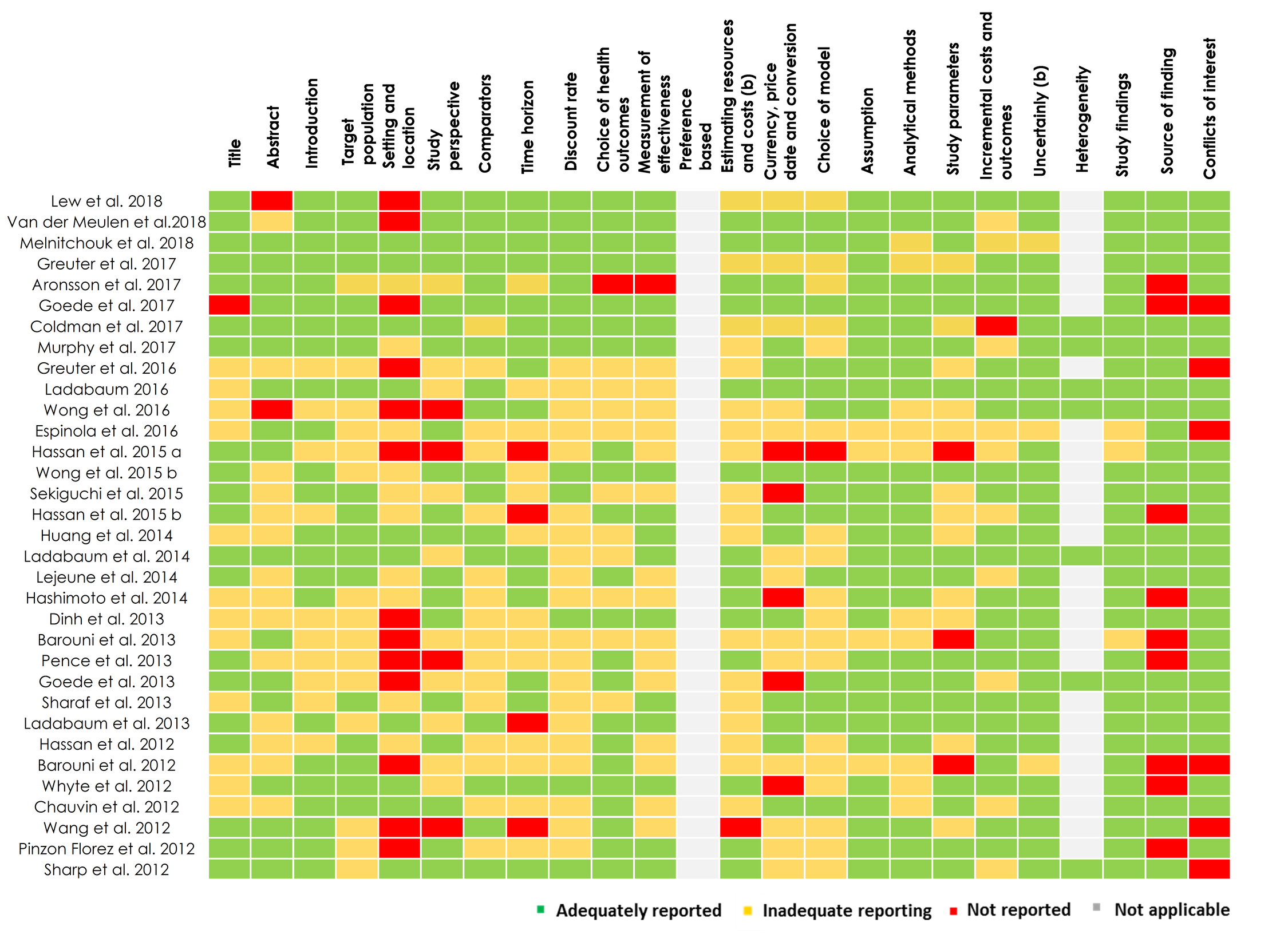

Supplement: S1 Fig — (TIF) [file pone.0227251.s006.tif]
